# Toward Personalized Theranostics: A Modular PET Platform for Antibody‐Based Imaging and Therapy

**DOI:** 10.1002/advs.76940

**Published:** 2026-08-20

**Authors:** Delphine Vivier, Megane Grah Kple, Julen Ariztia, Marion Leroux, Mathieu Moreau, Michael Claron, Maxime Cheveau, Adam Laouafa, Agnieszka Kownacka, Camille Petitot, John Simonet, Alexandre M. M. Dias, Sophie Poty, Charles Truillet, Bertrand Collin, Alexandra Oudot, Franck Denat

**Affiliations:** ^1^ CNRS ICMUB UMR 6302 Université Bourgogne Europe Dijon France; ^2^ Centre Georges‐François Leclerc‐Unicancer INSERM BioSanD US 58 Plateforme IMATHERA Université Bourgogne Europe Dijon France; ^3^ CEA CNRS Inserm BioMaps Université Paris‐Saclay Orsay France

**Keywords:** bischelate immunoconjugate, copper‐free click chemistry, modular antibody platform, PET/SPECT imaging, site‐specific conjugation, targeted therapy, theranostics

## Abstract

The expansion of personalized medicine is encouraging the development of platforms capable of supporting individualized imaging and therapeutic strategies. The combination of antibodies, with their intrinsic selectivity, and a modular molecular probe provides a strong foundation for such approaches. In this context, a versatile platform enabling positron emission tomography (PET) imaging followed by copper‐free click‐based functionalization is developed. A DFO–Lysine–DBCO probe is synthesized and site‐specifically coupled to trastuzumab, allowing the introduction of diverse payloads, including β‐ or α‐emitter chelators, a fluorophore, and a cytotoxic agent. Within this toolbox, the DFO/DOTAGA bischelate is selected for detailed evaluation. Radiolabeling confirms the selectivity of DFO and DOTAGA for ^89^Zr and ^177^Lu, respectively. PET imaging in mice bearing HCC1954 xenografts (HER2‐overexpressing, trastuzumab‐resistant) using [^89^Zr]Zr‐ss‐Tzb‐DFO‐DOTAGA and [^89^Zr]Zr‐ss‐Tzb‐DFO‐Lu(III)DOTAGA, in which DOTAGA is pre‐loaded with cold lutetium, enables clear tumor visualization and indicates that second‐chelator occupancy does not alter biodistribution. Despite unexpectedly lower internalization and tumor uptake, [^177^Lu]Lu‐ss‐Tzb‐DFO‐DOTAGA demonstrates significant antitumor activity with good tolerability in a preliminary therapeutic study. This modular platform, enabling site‐specific conjugation, provides a broadly adaptable route to generate multifunctional antibody constructs, offering a versatile foundation for integrating imaging, therapy, and payload customization within a single molecular scaffold.

## Graphical abstract

This modular, site‐specific antibody platform enables customizable conjugates for PET imaging and therapy. Interchangeable payloads – including radiometals, cytotoxics, and fluorophores – support adaptable, multimodal applications with limited off‐target effects. In vivo, the DFO/DOTAGA bischelate immunoconjugate provided clear tumor delineation in PET/SPECT imaging and demonstrated potent therapeutic benefit in nude mice bearing HCC1954 xenografts.

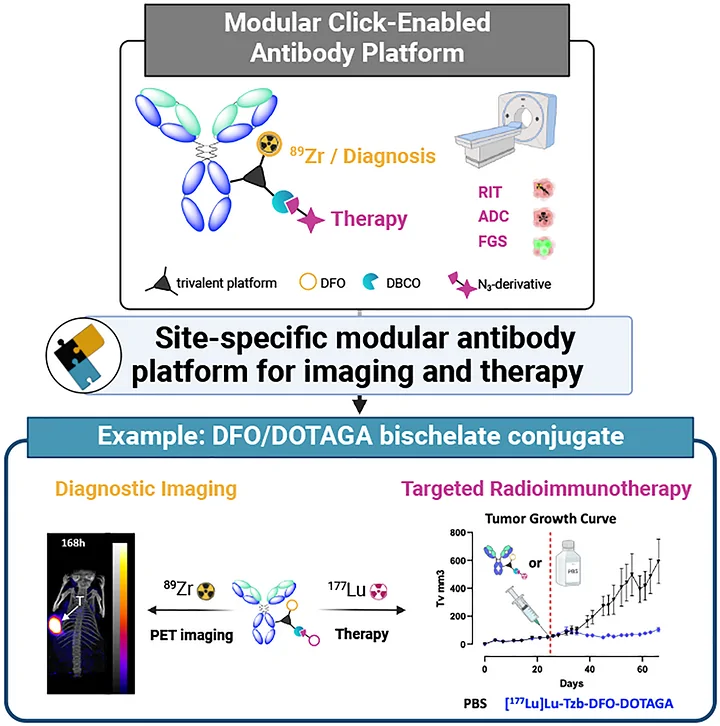

## 1 Introduction

Personalized medicine aims to tailor medical interventions to the biological characteristics of individual patients rather than relying on one‐size‐fits‐all approaches [1, 2]. Guided by biomarkers that predict therapeutic response and toxicity, this approach has reshaped oncology, improving outcomes while reducing unnecessary exposure to ineffective therapies. However, implementation remains challenging, particularly for targeted therapies whose efficacy depends on heterogeneous antigen expression across tumors and metastases [3]. Because tissue biopsies provide only localized information, there is a growing need for non‐invasive, quantitative tools capable of assessing whole‐body target expression to guide patient selection.

Theranostics – pairing molecular imaging with matched targeted therapies – has emerged as a powerful solution within precision oncology. PET imaging in particular offers high sensitivity, excellent quantification, enabling clinicians to identify appropriate candidates, optimize therapeutic dosing, and monitor treatment response. It can be associated with various targeted therapies. For example, an ongoing clinical trial is evaluating whether the PET imaging probe ^68^Ga‐MY6349 can predict the therapeutic efficacy of an anti‐TROP2 antibody‐drug conjugate (ADC) [4]. Although not yet in clinic, several groups, including ours, are also investigating preoperative PET imaging to select patients for fluorescence‐guided surgery (FGS) [5, 6, 7]. In parallel, targeted radionuclide therapy (TRT) has expanded beyond hematologic cancers to solid tumors with agents such as [^177^Lu]Lu‐DOTATATE and [^177^Lu]Lu‐PSMA‐617 now clinically available [8, 9].

However, most current theranostic systems rely on a dual‐agent strategy—one radiolabeled construct for PET imaging (companion diagnostic) and a second for therapy—which increases manufacturing costs and can introduce discrepancies in biodistribution when different payloads are used. To address these limitations, modular platforms allowing both diagnosis and therapy on a single molecular scaffold are of growing interest. In the TRT field, this concept has led to “convergence” radiopharmaceuticals, using the same vector‐chelator scaffold for imaging and therapy. Several examples demonstrate this emerging concept, including ^64^Cu/^67^Cu pretargeted radioimmunotherapy [10], dual ^64^Cu/^177^Lu PCTA‐cetuximab conjugates [11], and the ^89^Zr‐compatible chelator CHX‐A''‐DTPA [12]. However, such systems remain constrained by the limited number of true theranostic radiometal pairs or by challenges in achieving selective, stable coordination of different metals. Dual‐chelator systems offer a potential solution, but major obstacles remain, including compatibility issues—such as cross‐metal binding—lengthy synthetic routes that hinder practical translation, and limited versatility across different applications [13, 14].

To overcome these limitations, there is a need for a generalizable platform enabling the late‐stage installation of diverse functional payloads on a single antibody without altering its targeting properties. Ideally, such a system would allow incorporation of a radiometal for PET imaging alongside a therapeutic moiety—whether a radiometal for TRT, a fluorescent dye for optical surgery, or a cytotoxic agent for targeted drug delivery—enabling customization of the treatment plan from a single precursor construct. Site‐specific bioconjugation is crucial to ensure optimal in vivo performance, as it is known to improve homogeneity and reproducibility, ultimately enhancing biodistribution by increasing target affinity, stabilizing the conjugate, and reducing off‑target accumulation [15]. These benefits have been demonstrated in several preclinical models [16, 17], as well as in a recent first‐in‐human study showing improved lesion detection with a site‐specific HER2‐targeted immunoconjugate [18]. Among the available methods, microbial transglutaminase (MTGase) offers a simple, scalable approach to site‐specific modification through transamidation at conserved Fc glutamine residues [19]. Moreover, lysine – widely used in our group to produce MOnomolecular Multimodal Imaging Probes (MOMIPs) – is a natural substrate for this enzyme. As a result, it enables site‐specific conjugation without the need for introducing an additional functional group, thereby simplifying the synthesis process.

In this work, we describe a modular, site‐specific antibody platform that enables the late‐stage installation of diverse payloads through copper‐free click chemistry. We first synthesized a DFO–Lys–DBCO precursor and conjugated it site‐specifically on trastuzumab via MTGase. The DBCO handle was then used to introduce a broad panel of azide‐functionalized therapeutic modules, including α‐ and β‐emitter chelators, a fluorophore, and a cytotoxic agent. From this toolbox, we selected the first site‐specific DFO/DOTAGA bischelate immunoconjugate for detailed evaluation. We assessed its radiolabeling with ^89^Zr and ^177^Lu, stability in PBS and plasma, and in vivo performance in mice bearing trastuzumab‐resistant HER2‐positive HCC1954 xenografts. A small‐scale therapeutic study further explored its antitumor potential.

Our results demonstrate that this strategy provides a generalizable and highly adaptable foundation for personalized theranostics, enabling patient selection via PET imaging, targeted radionuclide therapy, fluorescent imaging, and drug delivery from a single antibody scaffold. The platform thus offers a powerful route to accelerate precision oncology through flexible, late‐stage antibody engineering.

## 2 Results

### 2.1 Design and Synthesis of the DFO‐Lys‐DBCO Probe

As outlined in the introduction, we aimed to develop a PET imageable probe suitable for both diagnosis and patient selection, which could be further functionalized via click chemistry to introduce a therapeutic moiety (Figure 1A, Figure S1). Since one of our next objectives was to incorporate therapeutic radioisotopes such as ^177^Lu and ^225^Ac, we ruled out the use of positron emitters like ^64^Cu, as co‐chelation or cross‐labeling between isotopes could occur. Instead, we selected ^89^Zr, since it is the most commonly used radionuclide for immunoPET [20], and DFO – the gold standard chelator for zirconium – is not known to chelate either ^177^Lu or ^225^Ac under standard protein radiolabeling conditions. For the click chemistry handle, we chose DBCO due to the wide commercial availability of azide‐bearing compounds capable of reacting with DBCO via strain‐promoted azide–alkyne cycloaddition (SPAAC). The DFO‐Lys‐DBCO probe was synthesized in four steps with an overall yield of 16% (Figure 1B). First, a peptide coupling was performed to attach DFO‐mesylate to commercially available Boc‐L‐Lys(Fmoc)‐OH **1**. After deprotection of the α‐amino group, a second peptide coupling with dibenzoazacyclooctyne‐carboxylic acid succinimidyl ester **3** introduced the clickable moiety. Final deprotection of the lysine side chain provided the ready‐to‐conjugate probe **4**.

**FIGURE 1:**
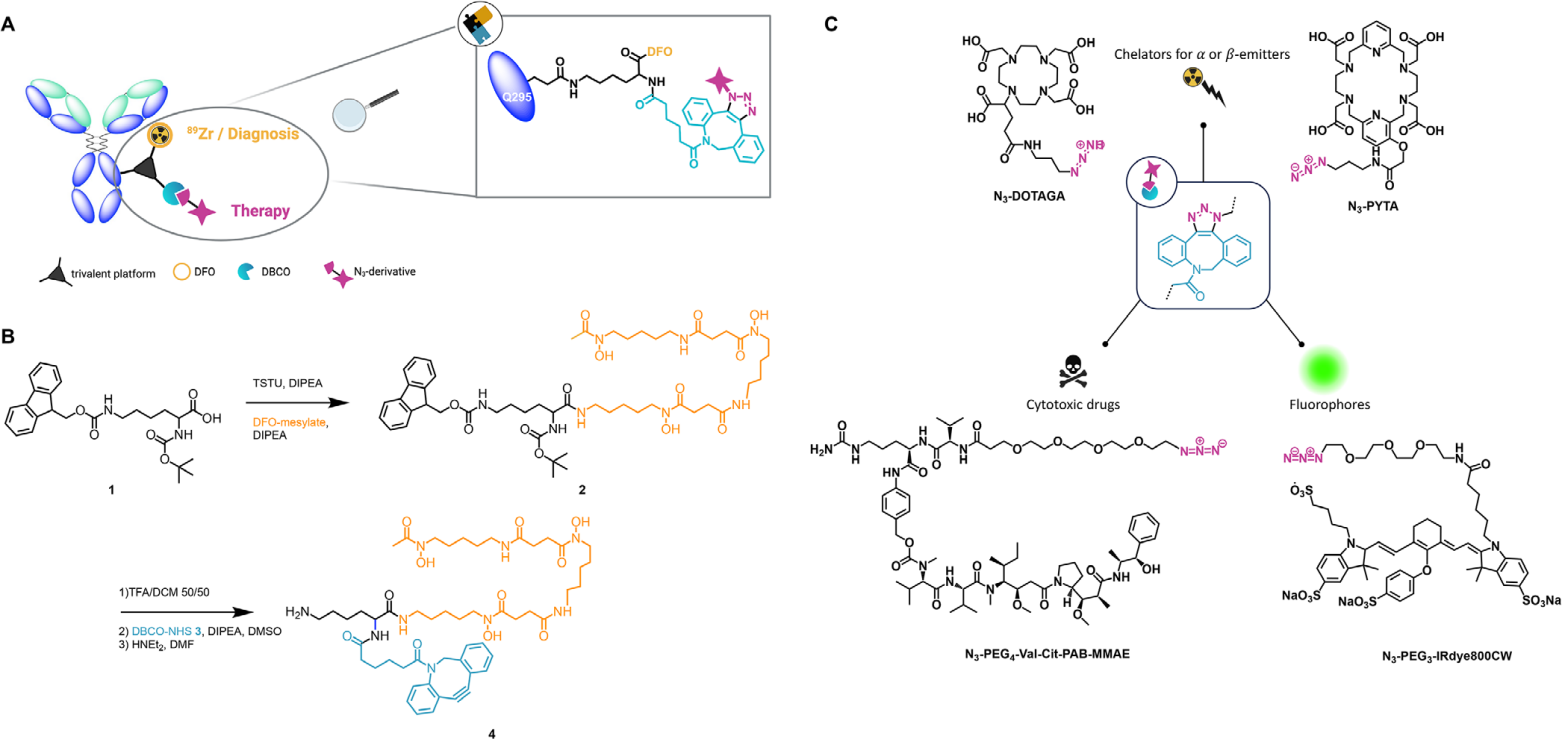
Design and versatility of a modular antibody platform. (A) Concept of the modular platform enabling PET imaging and late‐stage introduction of therapeutic payloads. (B) Synthesis of the clickable probe DFO‐Lys‐DBCO 4. (C) Strain‐promoted azide‐alkyne cycloaddition (SPAAC) with diverse payloads, including radiometal chelators, cytotoxic agents and fluorophores.

### 2.2 Site‐Specific Bioconjugation of Different Therapeutic Groups

To demonstrate the versatility of our modular platform, we next applied it to trastuzumab, an FDA‐approved monoclonal antibody widely used for the treatment of HER2‐positive breast cancer. HER2 is a well‐validated target for imaging and targeted therapy, and radiolabeled [^177^Lu]Lu‐trastuzumab has recently been evaluated in patients with metastatic disease [21], providing a clinically relevant model to assess the performance of our platform. Importantly, our strategy is not restricted to human IgG_1_: because MTGase enzyme recognizes a highly conserved sequence on the Fc region, it can be applied to most human IgG subclasses. Thus, the approach demonstrated here is broadly applicable to other antibodies and indications.

We then selected a panel of azide‐bearing therapeutic payloads to showcase the modularity of the conjugation strategy. For the first therapeutic radiometal, we chose ^177^Lu, which has become a leading isotope in TRT. To achieve stable complexation under mild conditions, DOTAGA was picked as the therapeutic chelator. As previously reported and corroborated by our preliminary results (Figures S2 and S3), DOTAGA does not effectively chelate ^89^Zr, nor does DFO bind ^177^Lu, thereby minimizing the risk of cross‐chelation and unintended metal complexation.

Given the increasing interest in emerging radionuclides such as ^225^Ac and ^161^ Tb – both of which have shown promising therapeutic profiles compared to ^177^Lu [22, 23, 24] – we also explored a PYTA derivative [25], a novel chelator well‐suited for actinium‐225 and terbium‐161 coordination. Importantly, PYTA does not bind zirconium, making it compatible with our theranostic strategy that requires orthogonality between the imaging and therapeutic components (unpublished data). Beyond chelators, we aimed to demonstrate the versatility of our platform by incorporating MMAE, a cytotoxic payload used in ADCs, and IRDye800CW, a near‐infrared fluorophore commonly used in fluorescence‐guided surgery.

The first step to obtain the bimodal immunoconjugate was to remove the glycan chains using the PNGaseF enzyme (Figure 2A). The resulting deglycosylated trastuzumab **5** was then conjugated to the lysine probe **4** via the enzymatic reaction with MTGase. This enzyme catalyzes the site‐specific transamidation between the side chain of glutamine 295 – located on the heavy chains – and the primary amine of our probe. Finally, the SPAAC between Tzb‐DFO‐DBCO **6** and the four azide derivatives **7 – 10** was performed to give the bimodal immunoconjugates ss‐Tzb‐DFO‐DOTAGA **11**, ss‐Tzb‐DFO‐PYTA **12**, ss‐Tzb‐DFO‐MMAE **13**, ss‐Tzb‐DFO‐IR800 **14**. The degrees of labeling (DOL) of all payloads were assessed via mass spectrometry (Figure 2B,C, Figures S4–S9). To ensure that conjugation did not alter the structural integrity of the antibody, its thermal stability and its inclination to form aggregates were assessed using the Prometheus Panta system (Figure 2D,E).

**FIGURE 2:**
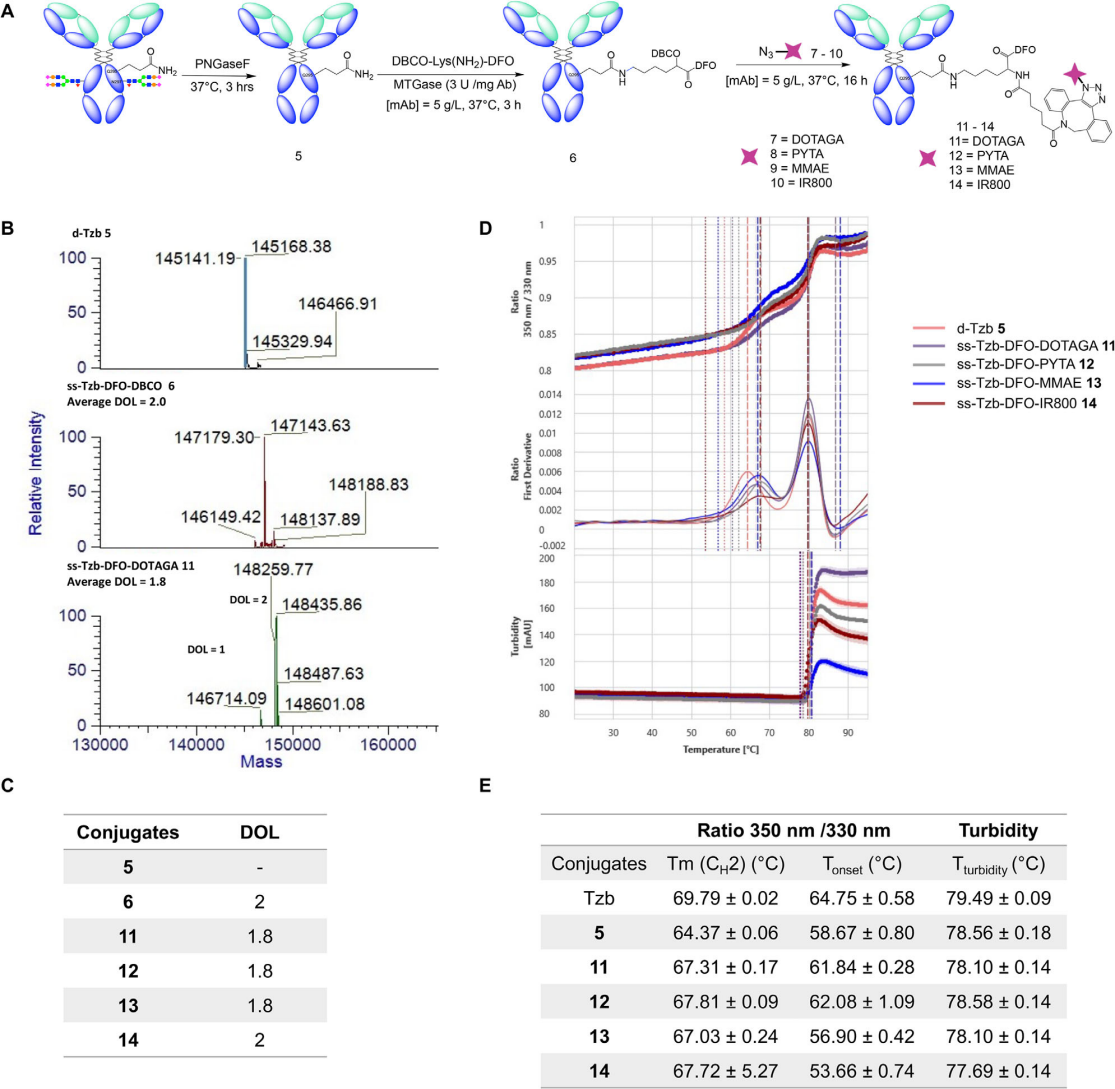
Preparation and biophysical characterization of the theranostic immunoconjugates 11–14. (A) Three‐step conjugation strategy: enzymatic deglycosylation 5, site‐specific attachment of DFO‐Lys‐DBCO 6, and strain‐promoted azide‐alkyne cycloaddition (SPAAC) with payloads 7‐10 to generate conjugates 11–14. (B) Deconvoluted mass spectra of the intermediate constructs, shown here for the DFO–DOTAGA series as a representative example, including deglycosylated trastuzumab 5, ss‐Tzb‐DFO‐DBCO 6, and ss‐Tzb‐DFO‐DOTAGA 11. (C) Degree of labeling (DOL) for all constructs. (D) Thermal stability analysis measured using a Prometheus PANTA (NanoTemper Technologies): intrinsic tryptophan fluorescence ratio at 330/350 nm (top), first derivative of the fluorescence signal to visualize melting transitions (middle), and turbidity to monitor aggregation (bottom). (E) Summary of thermal parameters, including melting temperature (Tm), unfolding onset temperature (T_onset_), and aggregation temperature (T_turbidity_).

As expected, deglycosylation of trastuzumab resulted in a decreased melting temperature (Tm) for the C_H_2 domain (69.79 ± 0.02°C for Tzb vs. 64.37 ± 0.06°C for deglycosylated Tzb **5,** Figure S10). Similarly, the onset of unfolding (T_onset_), indicating the beginning of the denaturation process, was also reduced, suggesting a loss of thermal stability (64.75 ± 0.58°C for Tzb vs. 58.67 ± 0.80°C for deglycosylated Tzb **5**). However, this destabilization did not lead to increased aggregation, as the onset temperatures for turbidity (T_turbidity_), marking the formation of aggregates, remained similar and appeared to correlate with the second transition (unfolding of the Fab fragment). Conjugation of the different therapeutic groups did not impact the unfolding profile as Tm were comprised between 67.03 ± 0.24°C and 67.81 ± 0.09°C and the T_turbidity_ ranged from 77.69 ± 0.14°C to 78.58 ± 0.14°C.

In addition, aggregate formation was also evaluated by size‐exclusion chromatography and was found to be below 4% for all conjugates (Figure S11).

In summary, we successfully developed an imageable and modular platform enabling the site‐specific conjugation of diverse therapeutic payloads onto trastuzumab. The resulting bimodal immunoconjugates retained excellent structural integrity and thermal stability, demonstrating that our enzymatic and click‐chemistry‐based approach is both efficient and broadly applicable.

### 2.3 DFO/Dotaga Bischelate Probe Evaluation

We then proceeded with a comprehensive evaluation of the DFO/DOTAGA bischelate immunoconjugate with a specific focus on how both the presence of the second chelator and the choice of radiometal influence in vivo biodistribution. To enable direct comparisons, we prepared a series of control immunoconjugates using the same enzymatic transglutaminase‐based conjugation method. These included two site‐specific monochelate conjugates – ss‐Tzb‐DFO **15** and ss‐Tzb‐DOTAGA **16** – as well as the clickable intermediate (ss‐Tzb‐DFO‐DBCO **6**) and a non‐radioactive (cold) bischelate conjugate ss‐Tzb‐DFO‐Lu(III)DOTAGA **17** (Figures S12–S14). The latter was prepared by performing a SPAAC reaction between ss‐Tzb‐DFO‐DBCO **6** and N_3_‐Lu(III)DOTAGA **18** (Figures S15 and S16).

As previously, the thermal stability of the different immunoconjugates was assessed using the Prometheus Panta system. All constructs displayed highly similar thermal unfolding profiles, with melting temperatures (Tm) ranging from 65.24 ± 0.11°C to 67.48 ± 0.24°C and turbidity transition temperatures (T_turbidity_) between 78.02 ± 0.00°C and 78.58 ± 0.14°C. The only noticeable destabilization was observed upon deglycosylation, consistent with our previous findings (Figure S17).

The binding affinities of Tzb, d‐Tzb **5**, ss‐Tzb‐DFO‐DBCO **6**, ss‐Tzb‐DFO‐DOTAGA **11** and ss‐Tzb‐DFO‐ Lu(III)‐DOTAGA **17** were determined by biolayer interferometry. All constructs exhibited affinities in the nanomolar range (Figure S18).

### 2.4 ^89^Zr Radiolabeling and Stability Studies

We performed ^89^Zr radiolabeling using ss‐Tzb‐DFO‐DBCO **6**, the bischelate ss‐Tzb‐DFO‐DOTAGA **11**, and the monochelate controls ss‐Tzb‐DFO **15** and ss‐Tzb‐DOTAGA **16**, to confirm the absence of cross‐chelation and assess the stability toward radiolysis. A specific activity of 60 MBq mg^−1^ was targeted, based on previous observations from our group indicating that radiolysis‐induced degradation may occur above this threshold when using NCS‐based linkers [26]. All DFO‐containing conjugates exhibited high radiolabeling efficiencies, with chelation yields exceeding 94% after 1 h, as determined by iTLC and radio‐HPLC. The presence of either the DOTAGA or DBCO moiety did not interfere with ^89^Zr complexation by DFO, and comparable specific activities were obtained for all three conjugates. As expected, the DOTAGA‐only conjugate (Tzb‐DOTAGA **16**) showed minimal incorporation of ^89^Zr (<3%), confirming the selectivity of DFO for zirconium under the applied radiolabeling conditions. To assess in vitro stability, radioconjugates were challenged with a 1000‐fold molar excess of EDTA or DFO (Figure S19), as well as incubated in PBS and human plasma at 37°C for up to seven days. Both competition assays indicated that the mono‐ and bischelate conjugates (**15** and **11**, respectively) displayed comparable resistance to transchelation. Similarly, [^89^Zr]Zr‐ss‐Tzb‐DFO‐DOTAGA and [^89^Zr]Zr‐ss‐Tzb‐DFO remained stable in PBS and plasma over the entire incubation period, with no evidence of radiometal release or radiolysis (Figure 3A). These results demonstrate that the presence of the second chelator does not compromise the stability of the ^89^Zr–DFO complex nor lead to nonspecific zirconium trapping under the labeling conditions used. In contrast, the precursor [^89^Zr]Zr‐ss‐Tzb‐DFO‐DBCO exhibited reduced stability, as indicated by the formation of high‐molecular‐weight species (>30%) detected by radio‐HPLC after 24 h, consistent with aggregation (Figure S20).

**FIGURE 3:**
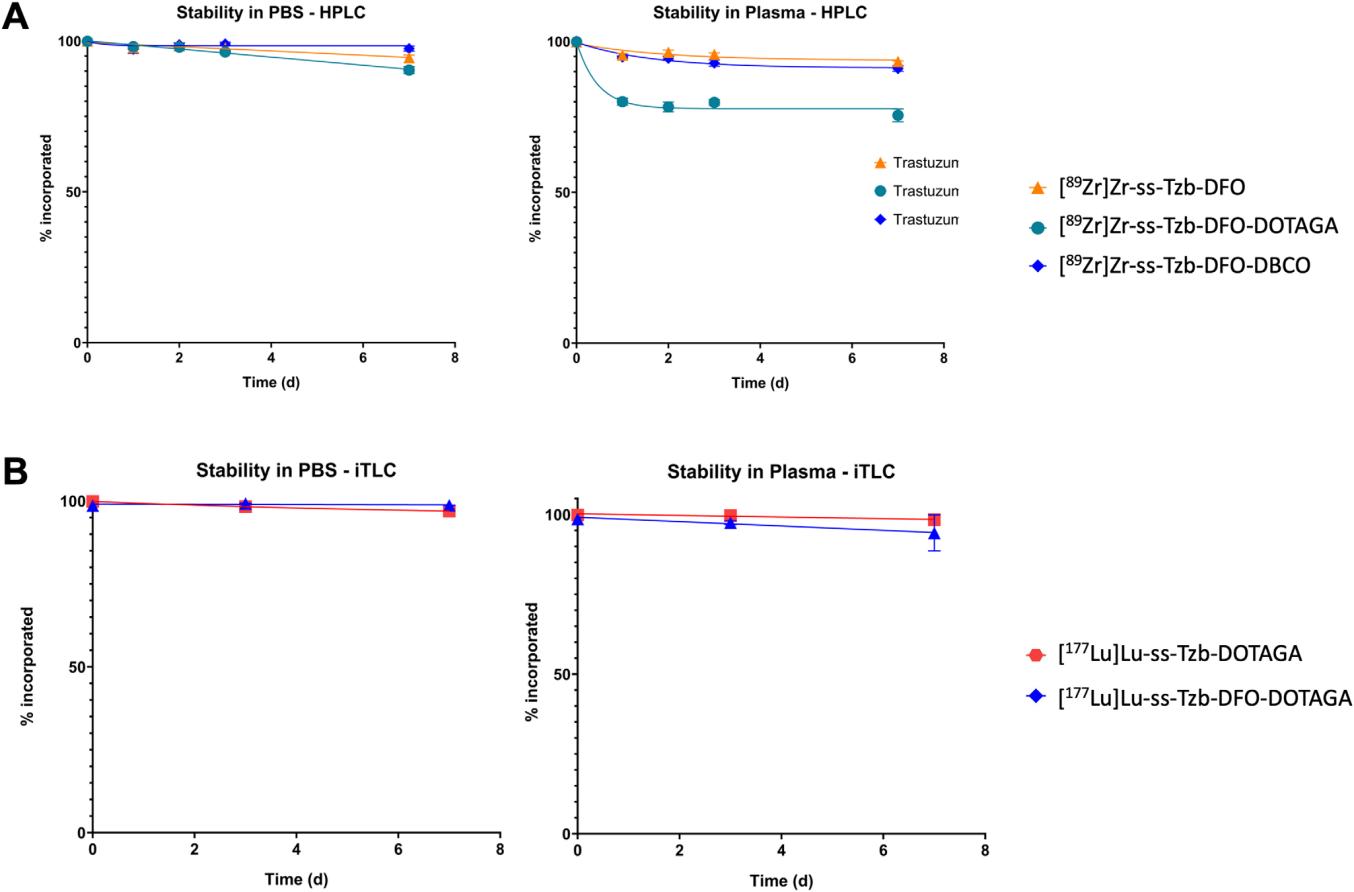
Stability of radiolabeled conjugates in PBS and plasma. A. Stability of [^89^Zr]Zr‐ss‐Tzb‐DFO, [^89^Zr]Zr‐ss‐Tzb‐DFO‐DBCO, and [^89^Zr]Zr‐ss‐Tzb‐DFO‐DOTAGA over a week at 37°C, measured by radio‐HPLC and expressed as % incorporated. B. Stability of [^177^Lu]Lu‐ss‐Tzb‐DOTAGA and [^177^Lu]Lu‐ss‐Tzb‐DFO‐DOTAGA over a week at 37°C, measured by iTLC and expressed as % incorporated. Error bars represent standard deviations (n = 3).

### 2.5 ^177^Lu Radiolabeling and Stability Studies

Similarly, ss‐Tzb‐DFO‐DBCO **6**, ss‐Tzb‐DFO‐DOTAGA **11**, and ss‐Tzb‐DOTAGA **16** were radiolabeled with ^177^Lu following standard protocols. The DOTAGA‐bearing conjugates demonstrated high radiolabeling efficiency, with incorporation rates exceeding 95%, and all achieved comparable specific activities of approximately 600 MBq mg^−1^. In contrast, no radiolabeling was observed for the DFO‐only precursor ss‐Tzb‐DFO‐DBCO **6**, as confirmed by both iTLC and radio‐HPLC analyses. As in previous experiments, the mono‐ and bischelate radioimmunoconjugates were incubated at 37°C for one week in PBS, human plasma (Figure 3B), and in the presence of a 1000‐fold molar excess of EDTA to assess their stability (Figure S21). Both [^177^Lu]Lu‐ss‐Tzb‐DFO‐DOTAGA and [^177^Lu]Lu‐ss‐Tzb‐DOTAGA exhibited identical stability profiles under all tested conditions, indicating that the presence of the second chelator does not affect the in vitro stability of the ^177^Lu‐DOTAGA complex.

### 2.6 In Vivo Behavior of ss‐Tzb‐DFO‐DOTAGA

To investigate the influence of the radiometal on in vivo behavior, we compared the biodistribution of [^89^Zr]Zr‐ss‐Tzb‐DFO‐DOTAGA, [^177^Lu]Lu‐ss‐Tzb‐DFO‐DOTAGA, and their corresponding monochelate conjugates in nude mice bearing subcutaneous HCC1954 xenografts. PET or SPECT imaging was performed, followed by *ex vivo* biodistribution analysis using gamma counting. In addition, a control group received [^89^Zr]Zr‐ss‐Tzb‐DFO‐Lu(III)‐DOTAGA to assess whether occupation of the second chelator influenced tissue distribution. Radiolabeling was performed at target specific activities of 80 MBq mg^−1^ for ^89^Zr and 300 MBq mg^−1^ for ^177^Lu (Figures S22 and S23). Compared with our earlier experiments conducted at 60 MBq mg^−1^, ^89^Zr incorporation was slower for both bischelate conjugates than for the monochelate ss‐Tzb‐DFO control. After 1 h, incorporation reached 97% for ss‐Tzb‐DFO, compared with 85% and 90% for ss‐Tzb‐DFO‐DOTAGA and ss‐Tzb‐DFO‐Lu(III)DOTAGA, respectively. Nevertheless, all three constructs displayed similar stability profiles in plasma and PBS, with 80–90% of radiometal retained after seven days (Figure S24). The ^177^Lu‐bischelate conjugate remained highly stable throughout the study, retaining >95% and >90% of activity in plasma and PBS, respectively (Figure S25).

Each mouse received 50 µg of antibody, corresponding to approximately 4 MBq for PET imaging or 15 MBq for SPECT imaging (Figure S26). Imaging was performed at 24, 72, and 168 h post‐injection. As evidenced by PET imaging and gamma counting, [^89^Zr]Zr‐ss‐Tzb‐DFO‐DOTAGA and [^89^Zr]Zr‐ss‐Tzb‐DFO‐Lu(III)DOTAGA exhibited similar biodistribution profiles, with strong tumoral uptake (158.60 ± 28.20 and 112.98 ± 19.38%ID g^−1^, respectively, at seven days p.i.) and limited off‐target signal (liver: 3.71± 0.17 vs 3.73± 0.26%ID g^−1^, spleen: 6.37 ± 0.18 vs 5.01 ± 1.44%ID g^−1^), behaving comparably to the monochelate control (Figure 4, Table S1). These data indicate that our bischelate conjugate can be used as a diagnostic tool and that the presence of the second chelator does not adversely affect biodistribution.

**FIGURE 4:**
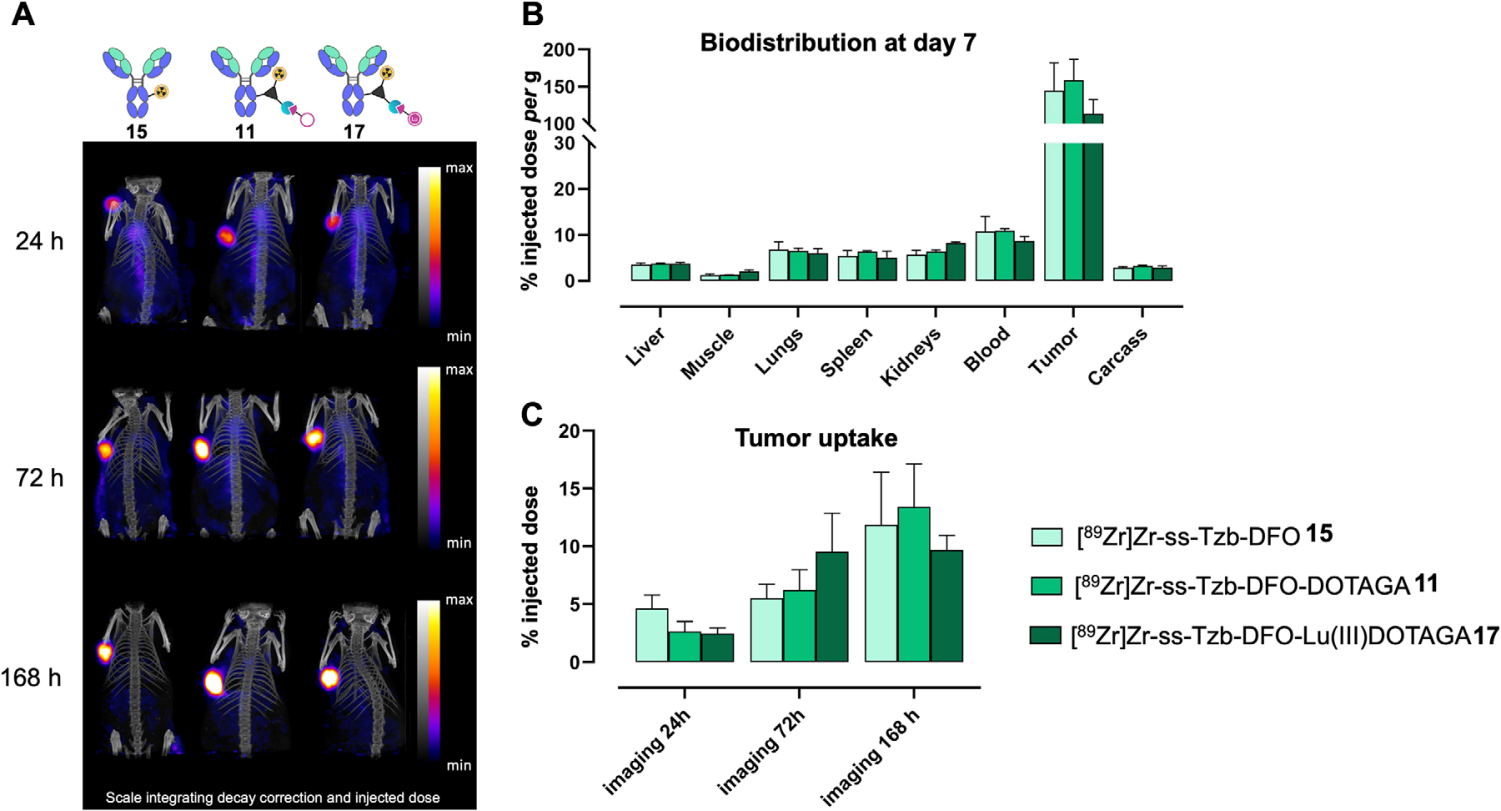
Influence of the second chelator—PET imaging and biodistribution of [^89^Zr]Zr‐ss‐Tzb‐DFO‐DOTAGA **11**, [^89^Zr]Zr‐ss‐Tzb‐DFO **15** and [^89^Zr]Zr‐ss‐Tzb‐DFO‐Lu(III)DOTAGA **17**. A. 3D maximum intensity projections of the different constructs. B. Ex vivo biodistribution (n = 3) of **11**, **15** and **17**. C. Pharmacokinetics of tumor uptake. Error bars represent standard deviations.

Moving to SPECT imaging, we observed a surprising phenomenon: although the tumors were clearly delineated for both the monochelate control [^177^Lu]Lu‐ss‐Tzb‐DOTAGA and the bischelate conjugate [^177^Lu]Lu‐ss‐Tzb‐DFO‐DOTAGA, the dual‐chelator construct showed markedly lower tumoral accumulation (69.31 ± 14.46 vs. 19.47 ± 0.99%ID g^−1^, respectively, at seven days p.i., Figure 5A, Figure S27, Table S2).

**FIGURE 5:**
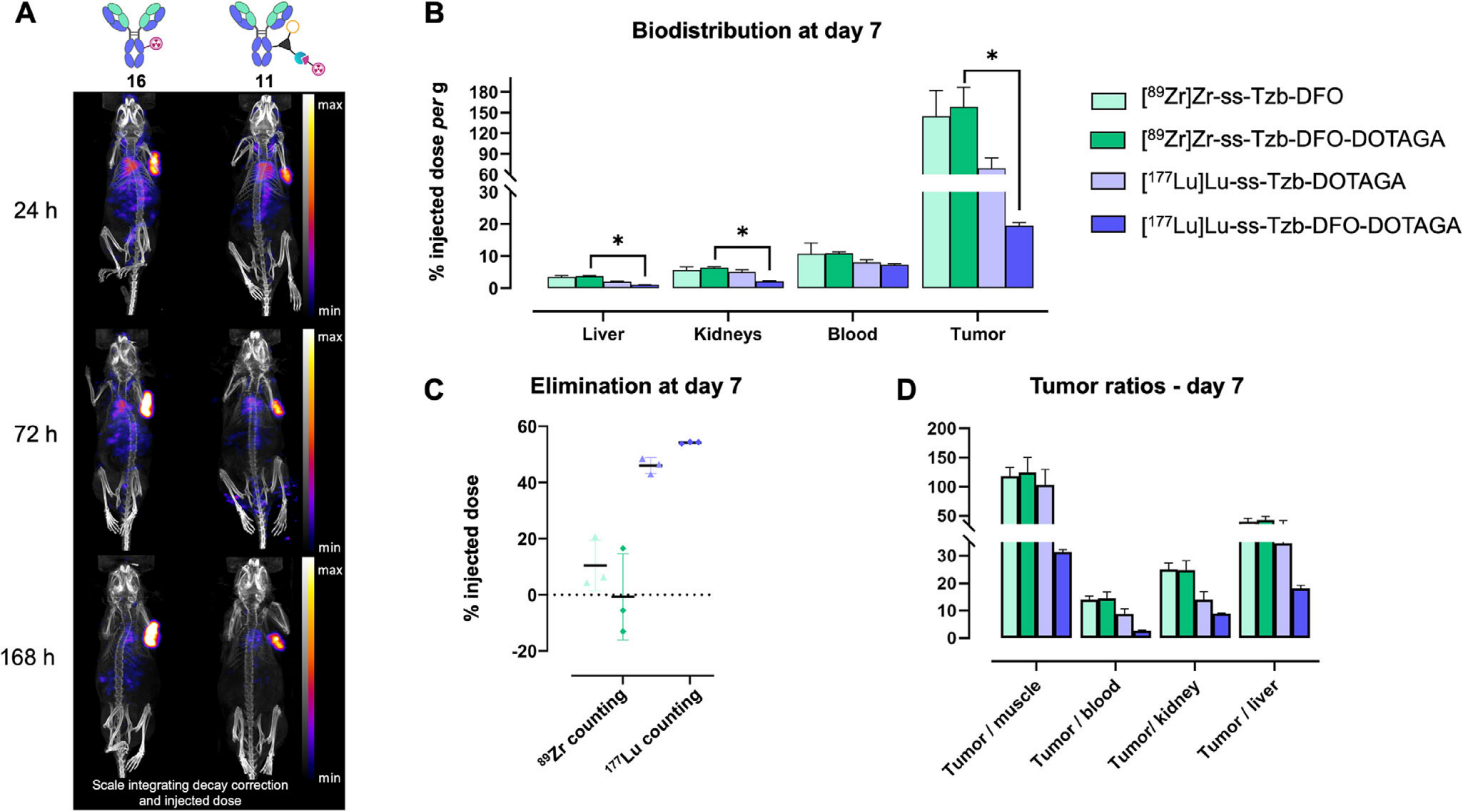
Influence of the radiometal on imaging and biodistribution. (A) SPECT imaging of [^177^Lu]Lu‐ss‐Tzb‐DFO‐DOTAGA **11**, [^177^Lu]Lu‐ss‐Tzb‐DOTAGA **16**. 3D maximum intensity projections of the different constructs. (B) Comparison across radionuclides. Ex vivo biodistribution of [^89^Zr]Zr‐ss‐Tzb‐DFO‐DOTAGA, [^89^Zr]Zr‐ss‐Tzb‐DFO [^177^Lu]Lu‐ss‐Tzb‐DFO‐DOTAGA, [^177^Lu]Lu‐ss‐Tzb‐DOTAGA at day 7 p.i. (n = 3). Differences between groups were analyzed using the Kruskal–Wallis test followed by Dunn's multiple comparison test. *p < 0.05. C. Elimination of the different constructs (% Injected Dose) at day 7 p.i. D. Tumor‐to‐organ ratios at day 7 p.i. Error bars represent standard deviations.

Comparing biodistribution profiles across radionuclides, we observed that both [^177^Lu]Lu‐conjugates accumulated less in the tumor than their zirconium‐labeled counterparts (Figure 5B). We also noted that the lutetium‐labeled conjugates were more rapidly eliminated, with ∼50% of the injected dose cleared after one week, compared to less than 20% for the zirconium‐labeled constructs (Figure 5C). However, tumor‐to‐organ ratios remained favorable – above 7 for all constructs except for the tumor‐to‐blood ratio of the ^177^Lu‐bischelate conjugate (2.68 ± 0.15, Figure 5D, Tables S3 and S4). Based on these results, we proceeded to the therapy study.

### 2.7 Therapy Study With [^177^Lu]Lu‐ss‐Tzb‐DFO‐DOTAGA

The therapeutic efficacy of [^177^Lu]Lu‐ss‐Tzb‐DFO‐DOTAGA was assessed in HCC1954 tumor–bearing mice. Because this cell line is resistant to trastuzumab, any antitumor effect could be solely attributed to the radioimmunotherapy [27]. A control group received only the vehicle (PBS), while the treated cohort received 6 MBq of [^177^Lu]Lu‐ss‐Tzb‐DFO‐DOTAGA. Body weight and tumor volume were monitored over a 7‐week period following injection (Figure 6, Tables S5–S7).

**FIGURE 6:**
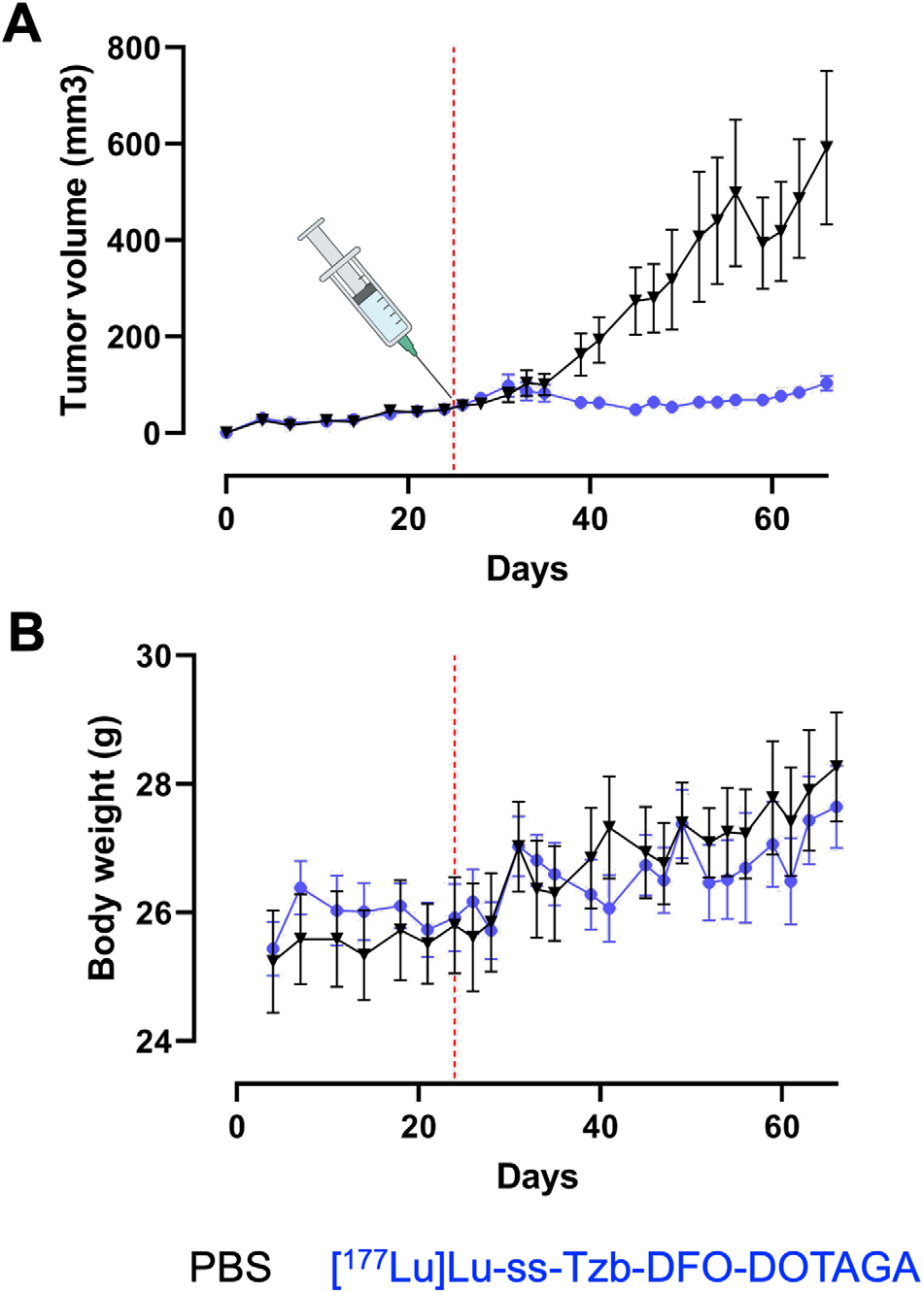
Therapeutic efficacy of [^177^Lu]Lu‐ss‐Tzb‐DFO‐DOTAGA in nude mice bearing HCC1954 xenografts. A. Tumor growth and B. Body weight were monitored for 7 weeks after injection of 6 MBq of [^177^Lu]Lu‐ss‐Tzb‐DFO‐DOTAGA (blue, n = 6) or PBS (black, n = 8 until day 56 then n = 7). Error bars represent the standard error of the mean (SEM). Tumor growth was analyzed using a mixed‐effects model with repeated measures, showing significant effects of time (p = 0.010), group (p = 0.0043), and time × group interaction (p = 0.026), indicating that Lu‐177 therapy significantly reduced tumor growth.

Tumor growth in the PBS control group increased steadily throughout the study, reaching an average volume of 592 mm^3^ by day 66. In contrast, mice treated with [^177^Lu]Lu‐ss‐Tzb‐DFO‐DOTAGA showed a slight reduction in tumor size around day 10 post‐treatment, followed by a prolonged period during which tumor size remained stable (between 60 and 70 mm^3^) until approximately day 61. Afterward, tumors began to increase slowly, reaching 103 mm^3^ by day 66.

Body weight regularly increased in both groups after treatment, indicating good tolerability of the treatment and absence of major systemic toxicity. No acute adverse effects were observed throughout the study. Despite its lower tumor accumulation compared with the ^89^Zr‐labeled conjugate, [^177^Lu]Lu‐ss‐Tzb‐DFO‐DOTAGA nonetheless exhibited clear antitumor activity in this trastuzumab‐resistant model.

## 3 Discussion

The combination of immunoPET – for lesion detection and patient stratification – and antibody‐based therapy (ADCs, RIT) offers a valuable and attractive theranostic approach. Yet, this approach often relies on separate companion diagnostics, and the difference between the chelator and the therapeutic moiety can lead to discrepancies in biodistributions [28].

In this work, we developed a versatile platform allowing both diagnostic imaging and therapy using a single immunoconjugate, thus eliminating the need for a companion diagnostic. Leveraging click chemistry, this modular probe can be functionalized with a variety of therapeutic moieties, providing a flexible and efficient toolkit for targeted cancer treatment.

Although more costly than random conjugation techniques, the transglutaminase strategy offers precise control over the conjugation process, yielding well‐defined and reproducible immunoconjugates. For clinical translation, one possible solution to mitigating costs and avoiding enzyme contamination in the final product is the use of immobilized mTGase, which enables enzyme recycling [29]. Another commonly cited limitation of this strategy is the need for antibody deglycosylation. Besides adding an extra step to the workflow, glycan removal may affect the stability of the resulting construct—particularly during long‐term storage. However, recent advances are addressing this concern. For example, a recent study demonstrated successful site‐specific modification of native antibodies using MTGase and short, positively charged peptides, eliminating the need for prior deglycosylation [30]. Together, these developments suggest that the key challenges associated with transglutaminase‐mediated conjugation are actively being addressed, further supporting its clinical applicability.

When comparing the biodistribution of the two bischelate conjugates, we were initially disappointed to observe the lower tumor accumulation of the therapeutic version. We suspect that the faster elimination of the ^177^Lu‐labeled bischelate conjugate may partly account for its reduced tumor uptake relative to the ^89^Zr analogue—an outcome that is not ideal for maximizing therapeutic delivery—and illustrates how even small chemical modifications can alter the in vivo behavior of a conjugate. As the bioconjugation site is located in proximity to the FcRn binding site, the receptor responsible for antibody recycling, we investigated whether radiometal‐dependent changes in FcRn binding could contribute to the in vivo discrepancies. For these studies, the corresponding non‐radioactive zirconium complex, ss‐Tzb‐Zr(IV)DFO‐DOTAGA **18**, was synthesized by complexation with cold zirconium (Figure S28). The affinities of the different constructs were measured by biolayer interferometry and we observed that deglycosylation reduced FcRn interaction whereas the introduction of the lysine platform restored binding to levels comparable to native Tzb (Figure S29). The click chemistry step appeared to negatively impact FcRn affinity but this effect was compensated following complexation with either Zr(IV) or Lu(III). Altogether, these findings suggest that altered FcRn binding alone is unlikely to explain the radiometal‐dependent differences in clearance.

Nonetheless, the accelerated elimination of the ^177^Lu constructs also resulted in lower exposure of dose‐limiting healthy tissues, which is beneficial in the context of radioimmunotherapy. For example, kidney uptake was only 2.18 ± 0.08%ID g^−1^for the ^177^Lu conjugate seven days post‐injection versus 6.33 ± 0.39%ID g^−1^for the ^89^Zr‐bischelate, and blood activity reached 7.27 ± 0.33%ID g^−1^compared with 10.91 ± 0.45%ID g^−1^, respectively.

To assess whether the probe design contributed to the observed pharmacokinetic differences, we prepared a “reversed” bischelate in which the DOTAGA chelator was directly attached to the lysine platform, while the DFO chelator was introduced via the click reaction. Following radiolabeling with ^177^Lu, stability assays in plasma and PBS were performed and analyzed by radio‐HPLC to compare the two architectures. Over the course of one week, no evidence was observed to suggest that the SPAAC linkage contributed to the altered in vivo behavior (Figure S30). However, the reversed bischelate was not evaluated in vivo, as we aimed to minimize animal use. We also explored radiometal‐dependent differences in hydrophobicity. Using hydrophobic interaction chromatography, we observed that ss‐Tzb‐Zr(IV)DFO‐DOTAGA exhibited greater hydrophobicity than its Lu counterpart (Figure S31).

Focusing on tumor activity, it is noteworthy that all three ^89^Zr conjugates continued to accumulate over a week, whereas both lutetium‐labeled conjugates appeared to reach a plateau at 72 h post‐injection — or even decline in the case of the bischelate. This suggests that radiometal‐dependent intracellular processing and residualization may also contribute to the lower tumor retention of the ^177^Lu conjugates. To explore this hypothesis, we performed internalization assays. Following radiolabeling, [^89^Zr]Zr‐ss‐Tzb‐DFO‐DOTAGA and [^177^Lu]Lu‐ss‐Tzb‐DOTAGA were incubated for 4 and 24 h with HCC1954 cells (Figure S32). As early as 4 h, the zirconium‐loaded construct displayed greater intracellular accumulation than its lutetium counterpart (∼5% versus ∼1% of total activity, respectively). This difference became even more pronounced after 24 h, with approximately 10% of [^89^Zr]Zr‐ss‐Tzb‐DFO‐DOTAGA internalized compared with about 1% for [^177^Lu]Lu‐ss‐Tzb‐DFO‐DOTAGA. In parallel, the membrane‐bound fraction remained relatively constant for the zirconium‐loaded conjugate (around 1%), whereas it progressively increased for the lutetium‐loaded construct (from 0.2% to more than 1%). These observations suggest that the lutetium‐loaded conjugate accumulates at the cell surface and undergoes substantially less internalization than its zirconium counterpart.

To determine whether this phenomenon was unique to the site‐specific construct, we additionally evaluated random bischelate conjugates prepared from either glycosylated (rd‐Tzb‐DFO‐DOTAGA **19**, Figure S33) or deglycosylated trastuzumab (d‐rd‐Tzb‐DFO‐DOTAGA **20**, Figure S34). Interestingly, only modest differences between radiometals were observed at 24 h post‐incubation for the glycosylated conjugates (∼7% versus ∼5% internalized activity for ^89^Zr and ^177^Lu, respectively), although the kinetics of internalization appeared slightly slower for [^177^Lu]Lu‐rd‐Tzb‐DFO‐DOTAGA. In contrast, the deglycosylated construct showed a marked reduction in internalization upon ^177^Lu complexation, with about 1% of the activity found intracellularly after 24 h *vs* about 6% for the ^89^Zr deglycosylated conjugate, consistent with the behavior observed for the site‐specific bischelate conjugate.

These findings suggest that impaired internalization of deglycosylated conjugates occurs specifically upon ^177^Lu complexation and may contribute to the in vivo discrepancies observed between the diagnostic and therapeutic bischelate conjugates. To our knowledge, such a radiometal complex‐dependent effect of antibody deglycosylation on cellular internalization has not been previously reported and warrants further mechanistic investigation. In particular, it will be interesting to determine whether this phenomenon is limited to ^177^Lu or extends to other radiometals coordinated by DOTAGA, and to investigate the impact of the nature of the chelator, which may also modify the charge and geometry of the radiometal complex. Likewise, evaluating other site‐specific conjugation methods that do not require deglycosylation – such as cysteine‐based conjugation strategies or the recently reported mTGase‐mediated modification of native antibodies developed by Wehrmüller et al. [30] – may help assess the respective contribution of radiometal coordination, glycosylation, and conjugation site to intracellular trafficking. Since receptor‐mediated endocytosis is generally accompanied by conformational rearrangements of the receptor–ligand complex, one possible hypothesis is that the combination of deglycosylation and ^177^Lu complexation subtly alters the structural or physicochemical properties of the conjugate in a manner that disfavors the internalization process. However, the molecular basis of this phenomenon remains unknown.

Although the pharmacokinetic differences prevent quantitative dosimetry extrapolation, the ^89^Zr bischelate remains useful for confirming tumor targeting and identifying patients most likely to benefit from the therapeutic version. Importantly, despite the lower tumor uptake of the ^177^Lu bischelate compared with its diagnostic counterpart, a significant therapeutic effect was still observed in the HCC1954 model. Nevertheless, further evaluation in additional tumor models and with appropriate control groups, including isotype‐matched controls or blocking experiments, will be necessary to establish whether these findings extend to other settings.

In addition to its versatility, the clickable probe offers significant advantages for radiochemistry. Radiolabeling proteins is generally more challenging than labeling small molecules or peptides, particularly due to the temperature sensitivity of proteins, which cannot be heated above 37°C. Our two‐step strategy overcomes this limitation: the azido‐bearing chelator can first be radiolabeled under harsh conditions, and then conjugated to the protein via a mild click reaction. This approach enables the preparation of radiolabeled immunoconjugates that would otherwise be difficult to obtain, such as those involving ^225^Ac chelated with DOTAGA which typically require high‐temperature conditions incompatible with antibodies [31]. Additionally, although overnight incubation was initially selected as a robust condition to ensure complete conversion at low protein concentration, we observed that the maximal DOL was already reached after approximately 2 h during the SPAAC conjugation step with DOTAGA–N_3_. This result further supports the applicability of this strategy to the preparation of radioimmunoconjugates.

Beyond the specific bischelate evaluated here, the modular design of the platform inherently supports the interchangeable use of different payload classes. Therefore, it enables treatment adjustments if clinical response is insufficient or if resistance emerges. While this flexibility was not explored in vivo in the present study, it represents an important conceptual advantage of the approach and aligns with the need for adaptable, imaging‐guided treatment strategies in personalized oncology. However, it is important to note that our lysine platform could be further optimized to address the stability limitations observed for the DBCO‐bearing intermediate. One possible explanation is the nature of the click moiety itself: DBCO is relatively hydrophobic and conformationally constrained, which may favor intermolecular interactions and contribute to aggregation. Several strategies could be considered to mitigate this issue, including replacement of DBCO with alternative bioorthogonal partners such as BCN or the introduction of PEG spacers to improve hydrophilicity and reduce intermolecular association. Importantly, this instability was only observed at the intermediate stage, whereas the final clicked immunoconjugates remained stable over at least one week. From a manufacturing perspective, the impact of intermediate instability could also be minimized by limiting storage time and performing the click‐conjugation step shortly after intermediate preparation.

Finally, this strategy opens a path to pretargeted radioimmunotherapy [32]. By replacing the SPAAC pair with the trans‐cyclooctene (TCO) / tetrazine ligation, it would be possible to perform PET imaging first to identify patients most likely to benefit from subsequent radioimmunotherapy. After imaging‐based selection, a small tetrazine‐bearing DOTAGA derivative could then be injected to deliver the therapeutic radionuclide selectively to the prelocalized TCO‐modified antibody at the tumor site.

## 4 Conclusions

In this study, we present a modular antibody‐based theranostic platform designed to combine molecular imaging, patient stratification, and targeted therapy within a single, well‐defined construct. By combining site‐specific bioconjugation with a bioorthogonal click‐chemistry strategy, we established a PET‐enabling scaffold that can be functionalized at a late stage with a variety of therapeutic or diagnostic payloads, including radionuclides for targeted radionuclide therapy, fluorophores for fluorescence‐guided surgery, or cytotoxic drugs for antibody–drug conjugates.

As a proof of concept, we developed the first site‐specific DFO/DOTAGA bischelate immunoconjugate and evaluated its in vivo behavior in a HER2‐positive breast cancer xenograft model. This construct allowed successful tumor imaging and demonstrated therapeutic efficacy following ^177^Lu labeling. Although differences in pharmacokinetics between the diagnostic and therapeutic radioconstructs prevent direct dosimetric extrapolation, the results highlight the value of this approach for confirming tumor targeting, selecting suitable candidates, and delivering targeted therapy. Moreover, our results suggest that the radiometal‐complex may influence the intracellular fate of deglycosylated immunoconjugates, an aspect that should be considered in the design of future theranostic antibodies.

Importantly, the two‐step modular design provides a general solution for radiolabeling biomolecules that are incompatible with conventional radiochemistry due to harsh labeling conditions. Furthermore, by adapting the bioorthogonal chemistry to alternative click pairs such as trans‐cyclooctene and tetrazine, this platform could be readily extended to pretargeted radioimmunotherapy strategies, a strategy currently under investigation in our laboratory.

Overall, this work establishes a modular theranostic scaffold capable of supporting PET imaging and multiple therapeutic modalities from a single molecular probe. Such versatility addresses key challenges in theranostic development and represents a significant step toward truly personalized, multimodal cancer management.

## 5 Experimental section

### 5.1 Probe Synthesis

Details of the probe synthesis and characterization can be found in the supplemental data, as well as general remarks about reagents and instruments.

### 5.2 Bioconjugation

Detailed protocols for deglycosylation and bioconjugation of each immunoconjugate are provided in the supplemental data.

#### 5.2.1 Enzymatic conjugation

A solution of deglycosylated trastuzumab **5** (1 equiv.) was mixed with DFO‐Lys‐DBCO **4** (15 equiv., solution stock at 60 mmol L^−1^ in PBS). Antibody concentration was adjusted to 5 g L^−1^ with phosphate buffered saline (PBS, pH 7.4). The reaction mixture was incubated at 37°C for 3 h after which the crude product was purified by size exclusion chromatography (Sephadex G‐25 M, PD‐10 column, GE Healthcare). The eluted fractions were concentrated using centrifugal filter units with a 50Dka molecular weight cut off (Amicon Ultra 2 mL Centrifugal Filtration Units, Millipore, Merck.) and phosphate buffered saline (PBS, pH 7.4) to obtain ss‐Tzb‐DFO‐DBCO **6**. The degree of labeling was estimated by mass spectrometry.

#### 5.2.2 Click chemistry

A solution of ss‐Tzb‐DFO‐DBCO **6** (1 equiv.) was mixed with the N_3_‐derivative (10 equiv. per DBCO, solution stock at 30 mmol L^−1^ in PBS). Phosphate buffered saline (PBS, pH 7.4) was added to achieve a final antibody concentration of 5 g L^−1^. After stirring the reaction at room temperature overnight, purification was carried out using a Protein A column (HiTrap MabSelect Xtra, Cytiva). Mass spectrometry was used to quantify the degree of labeling.

### 5.3 Thermal Stability Studies

The structural and thermal stability of trastuzumab derivatives were assessed using a Prometheus Panta instrument (NanoTemper Technologies), which enables simultaneous monitoring of nano‐differential scanning fluorimetry (nanoDSF), turbidity, and dynamic light scattering (DLS). Samples were diluted to 1 g L^−1^ in phosphate‐buffered saline (PBS, pH 7.4) and 10 µL of each sample were loaded into high‐sensitivity glass capillaries (NanoTemper Technologies, Cat.# PR‐C002). Data were acquired using Panta Control version1.10.2 and processed by Panta Analysis version1.10.2.

Thermal unfolding profiles were recorded from 20°C to 95°C using a linear heating rate of 1°C min^−^^1^. Intrinsic tryptophan fluorescence was excited at 280 nm, and emission intensities were measured at 330 nm and 350 nm. The fluorescence ratio (F_350_/F_330_) was calculated, and its first derivative was used to determine the melting temperature (Tm), corresponding to the major unfolding transition. Turbidity and DLS data were acquired simultaneously using back‐reflection optics and a 405 nm laser, respectively.

The Prometheus Panta Control Software automatically determined the temperature of aggregation onset (T_turbidity_), defined as the temperature at which a two‐state fit of the turbidity curve deviated from the baseline by more than 0.5%. All measurements were performed in triplicate, and the mean ± SD values of Tm, T_onset_ and T_turbidity_ were reported.

### 5.4 Size‐Exclusion Chromatography

SEC analyses of the different immunoconjugates were performed using an Arc Premier liquid chromatography Systeme (Waters) equipped with a MaxPeak Premier Protein Size‐Exclusion Chromatography column (250 Å, 2.5 µM, 4.6×300 mm, Waters). Phosphate‐buffered saline (PBS) was used as the mobile phase at a flow rate of 0.2 mL min^−1^.

### 5.5 Hydrophobic Interaction Chromatography

HIC analyses of ss‐Tzb‐DFO‐DOTAGA, ss‐Tzb‐Zr(IV)DFO‐DOTAGA and ss‐Tzb‐DFO‐Lu(III)DOTAGA were performed using an UltiMate 3000 chromatography Systeme (Thermo Fisher Scientific) equipped with a MabPac HIC‐butyl column (5 µM, 4.6×10 mm, Thermo Fisher Scientific). Phosphate‐buffered saline (PBS) was used as the mobile phase at a flow rate of 1 mL min^−1^.

### 5.6 Biolayer Interferometry

The affinity of trastuzumab derivatives for recombinant human HER2 was studied by biolayer interferometry (BLI) on a Sartorius Octet R2 system. All experiments were performed at 20°C with 1000 rpm shaking. High Precision Streptavidin 2.0 (Sartorius) biosensors were employed. Biotinylated human HER2 (ACROBiosystems) and trastuzumab derivatives were used as the ligand and analytes respectively. PBS was used as control. HER2 was diluted in PBS pH 7.4 to a concentration of 20 nM. Biosensors were first dipped into wells containing PBS for 10 min. The loading time of HER2 on the biosensors was 300 s. Biosensors were washed with PBS during 120 s before the association step. Association time of trastuzumab to HER2 was set to 90 s, and dissociation time was 300 s in PBS. Raw sensorgrams were fitted using a 1:2 bivalent analyte model in Octet data analysis HT software v 12.2. Data were corrected with an alignment of the Y axis to the average of baseline step, an alignment of the inter‐step to the association step and a Savitzky‐Golay fittering.

To minimize avidity effects and promote 1:1 kinetic interaction, the affinity between murine FcRn and the trastuzumab derivatives was measured using a reverse assay orientation. Trastuzumab derivatives were immobilized on FAB2G biosensors and mFcRn was used as the analyte. All experiments were performed at 20°C with 1000 rpm shaking. PBS was used as control. Immunoconjugates were diluted in PBS pH 7.4 to a concentration of 6.25 µg/ml. Biosensors were first dipped into wells containing PBS for 10 min. The loading time of immunoconjugate on the biosensors was 300 s. Biosensors were washed first with PBS during 30 s then for 210 s with a solution containing PBS and 0.05% Tween‐20 pH 6.0 as the association step was performed under acidic condition (interaction pH‐dependent). Association time of mFcRn was set to 600 s, and dissociation time was 60 s in PBS pH 6.0. Raw sensorgrams were fitted using a 1:1 monovalent analyte model in Octet data analysis HT software v 12.2. Data were corrected with an alignment of the Y axis to the average of baseline step, an alignment of the inter‐step to the association step and a Savitzky‐Golay fittering.

### 5.7 Radiolabeling Procedures

#### 5.7.1 Zirconium‐89

Immunoconjugates Were Radiolabeled With Zirconium‐89 (Revvity, Amsterdam) Using Standard Conditions. Briefly, the Conjugates, pH Adjusted to 7.4 With HEPES 1 M, Were Incubated for 1 h at 37°C in a Medium Containing [^89^Zr][Zr‐(Oxalate)_4_]^4−^ That Had Been Neutralized Using 2 M Na_2_CO_3,_ Targeting Specific Activity Around 60 or 80 MBq mg^−1^. Purification of the [^89^Zr]Zr‐conjugates Was Performed With Amicon Ultrafiltration Tubes (Millipore, Merck, Cutoff: 30 kDa). Radiochemical Purity of [^89^Zr]Zr‐conjugates Was Assessed by Instant Thin Layer Chromatography Performed on Glass Microfiber Strips Pre‐coated With Silicic Acid (iTLC‐SA, Agilent Technologies) and Eluted With 0.1 M EDTA, pH 5.0. Detection Was Performed on an AR‐2000 Radio‐TLC Plate Reader (Bioscan Inc.). Size‐exclusion High‐performance Liquid Chromatography (HPLC), Coupled to UV and Gamma Detection (Bioscan Inc., Counting Time: 1 Min), Was Employed to Confirm Radiochemical Purity. Prior to Injection, Antibody Radioconjugates Were Diluted in PBS to 0.5 G L^−1^

#### 5.7.2 Lutetium‐177

Immunoconjugates Were Radiolabeled With Lutetium‐177 (Itm, Munich) Using Standard Conditions. Briefly, Solutions of Antibody Conjugates in NH_4_OAc 1 M pH 5.4, Were Incubated With ^177^Lu for 1 h at 37°C, 300 Rpm to Reach a Specific Activity of 300 or 600 MBq mg^−1^. If Necessary, Purification of the [^177^Lu]Lu‐conjugates Was Performed With Amicon Ultrafiltration Tubes (Millipore, Merck, Cutoff: 30 kDa) After the Addition of DTPA 4 mM (0.09:1 (v/v) Ratio Relative to the Solution Volume). Radiochemical Purity of [^177^Lu]Lu‐conjugates Was Estimated by Instant Thin Layer Chromatography Performed on Glass Microfiber Strips Pre‐coated With Silica Gel (iTLC‐SG, Agilent Technologies), Eluted With 0.1 M Sodium Citrate pH 5.0 and Analyzed on an AR‐2000 Radio‐TLC Plate Reader (Bioscan Inc.). Radiochemical Purity Was Confirmed by Size‐exclusion High‐performance Liquid Chromatography (HPLC) Coupled With a UV and Gamma‐counter (Bioscan Inc., Counting Time: 1 Min). Prior to Injection, Antibody Radioconjugates Were Diluted in PBS to 0.5 G L^−1^

### 5.8 Stability Studies

#### 5.8.1 Zirconium‐89

After Radiolabeling, Solutions of Antibody Radioconjugates in HEPES 1 M Buffer (10 µL) at Specific Activities Around 60 and 80 MBq mg^−1^ Were Transferred to 90 µL of PBS, PBS + 1000 Equivalent of EDTA, or human Serum (PAN BIOTECH). Stability Study Solutions Were Incubated at 37°C for up to 7 Days. At Appropriate Time Points, 2–10 µL of the Solution Were Spotted on an iTLC Sheet for Analysis of the Percentage of ^89^Zr Activity Still Coordinated by the Chelator

#### 5.8.2 Lutetium‐177

After Radiolabeling, Solutions of Antibody Radioconjugates in NH_4_OAc 1 M Buffer (10 µL) at Specific Activities Around 300 and 600 MBq mg^−1^ Were Transferred to 90 µL of PBS, PBS + 1000 Equivalent of EDTA, or human Serum (PAN BIOTECH). Stability Study Solutions Were Incubated at 37°C for up to 7 Days. At Appropriate Time Points, 2–10 µL of the Solution Were Spotted on an iTLC Sheet for Analysis of the Percentage of ^177^Lu Activity Still Coordinated by the Chelator

### 5.9 Cell Culture

The human breast cancer cell line HCC1954 HER2/neu+ (ATCC CRL‐2338, ATCC; RRID: CVCL_1259) was maintained in RPMI‐1640 medium containing 10% foetal bovine serum (FBS) at 37°C in a 5% CO_2_ incubator.

### 5.10 Internalization Assay

The in vitro internalization of ^89^Zr‐ and ^177^Lu‐labeled trastuzumab‐based radioconjugates was assessed in HCC1954 HER2/neu+ cells. Cells were seeded in 24 well plates at 250,000 cells per well and incubated overnight in complete RPMI 1640 medium. Then, cells were washed with PBS and incubated with each radioconjugate at a final concentration of 10 nM in serum‐free medium. Non‐specific binding was evaluated in parallel using a 100‐fold molar excess of unlabeled trastuzumab. The evaluated compounds included [^89^Zr]Zr‐ss‐Tzb‐DFO‐DOTAGA (0.37 µg, 30 kBq), [^177^Lu]Lu‐ss‐Tzb‐DFO‐DOTAGA (0.37 µg, 110 kBq), and the corresponding randomly conjugated DFO/ DOTAGA trastuzumab analogues prepared from deglycosylated or glycosylated trastuzumab (0.37 µg, 30 kBq of ^89^Zr or 110 kBq of ^111^In). After 4 or 24 h at 37°C, plates were placed on ice to stop internalization. Cells were washed with ice‐cold PBS, and the membrane‐bound fraction was recovered using ice‐cold acidic glycine/NaCl buffer (0.05 M glycine, 0.15 M NaCl, pH 2.8). The remaining internalized fraction was obtained after cell lysis with 1 M NaOH. Radioactivity in each fraction was measured using a Wizard 1480 gamma counter (PerkinElmer, France).

### 5.11 Animal Model

All procedures involving animals were carried out following preclinical ethical standards and received approval from the local ethical committee (C2EA Grand Campus, project #47968, Dijon, France). A total of 10 × 10^6^ HCC1954 cells were administered subcutaneously in the right flank using a 27G needle (total volume of 100 µL in RPMI 1640 medium without foetal bovine serum FBS) of immunodeficient 8‐week‐old female Swiss Nude mice (Crl:NU(Ico)‐*Foxn1^nu^*, Charles River Laboratories, Saint Germain Nuelles, France, RRID: IMSR_CRL:NU). Our study exclusively examined female mice because the disease modeled (breast cancer) is mainly relevant in females. All cell injections were performed under anaesthesia with 2% of isoflurane. Animals were housed in pathogen‐free conditions at 22°C and 55% relative humidity, on a controlled 12 h inverted light‐dark cycle, with ad libitum access to standard diet and mineral water. Tumor growth was measured three times per week using callipers, and tumor volume (TV) was calculated using the formula TV = (x*y^2^)/2 where x represents the largest tumor diameter and y the corresponding perpendicular diameter.

### 5.12 Biodistribution Studies

Eighteen HCC1954 tumour‐bearing mice were randomized into six groups of n = 3 mice at 24 days post‐implantation. Three groups of mice received an intravenous (i.v.) dose of 50 µg/3 MBq/100 µL of either [^89^Zr]Zr‐Tzb‐DFO or [^89^Zr]Zr‐Tzb‐DFO‐DOTAGA or [^89^Zr]Zr‐Tzb‐DFO‐Lu(III)DOTAGA, and two groups of mice received an i.v. dose of 50 µg/15 MBq/100 µL of either [^177^Lu]Lu‐Tzb‐DOTAGA or [^177^Lu]Lu‐Tzb‐DFO‐DOTAGA. Imaging was performed for all mice at 24, 72 and 168 h post‐i.v. injection under anesthesia (1.5%–2% isoflurane).

PET‐CT images acquisitions were performed using a BioPET/CT (Sedecal, Spain). A CT scan was obtained (150 µA, 45 kV, 360 projections) and followed by a static PET imaging (46 mm axial FOV, 250–700 keV).

SPECT‐CT images acquisitions were performed using NanoSPECT/CT small animal tomographic gamma camera (Mediso Ltd., Budapest, Hungary formerly Bioscan, USA). CT acquisitions (45 kV, 13 mAs) were first acquired during 4–6 min, followed by helical SPECT acquisitions with 70–90 s per projection frame. Lutetium‐177 photopeaks (56 keV, 113 and 208 keV) were used with a 20% wide energy window. The PET/CT and SPECT/CT reconstructions were performed using image processing software provided respectively by Bioscan Inc. and Sedecal. The PET/CT and SPECT/CT fusion images were obtained using the Vivoquant software (Invicro, USA).

Mice were euthanized after the last imaging. Organs were collected for gamma counting (Wizard 1480, PerkinElmer, Villebon‐sur‐Yvette, France).

### 5.13 Therapy Study

Sixteen HCC1954 tumour‐bearing mice were randomized into two groups of n = 8 mice at 24 days post‐implantation. The first group of mice received one i.v. injection of vehicle (PBS 1x), the second group of mice received one i.v. dose of 50 µg/6 MBq/100 µL of [^177^Lu]Lu‐Tzb‐DFO ‐DOTAGA. Two mice were excluded from group 2 due to injection issues. The animals were then monitored for tumor volume and body weight 3 times per week during 7 weeks. Mice were euthanized when tumour volumes exceeded ethical limits (i.e. > 1500 mm^3^) or after 7 weeks of follow‐up.

### 5.14 Statistical Analysis

Statistical Analyses were performed using GraphPad Prism software (v10.6.1). Biodistribution differences across radionuclides were analyzed using the Kruskal–Wallis test followed by Dunn's multiple comparisons test. Tumor growth data were analyzed using a mixed‐effects model (REML) with repeated measures, with time as the within‐subject factor and treatment group as the between‐subject factor. The model accounts for the repeated measurements on individual animals (n = 6–8 per group). Post hoc comparisons were performed with Sidak correction for multiple testing, and statistical significance was set at p < 0.05. A Geisser‐Greenhouse correction was used. Data are presented as mean ± standard deviation (SD) unless otherwise stated.

### Author Contributions

**Delphine Vivier**: conceptualization, methodology, project administration, investigation, supervision, formal analysis, visualization, writing – original draft, writing – review and editing. **Megane Grah Kple**: investigation, writing – review and editing. **Julen Ariztia**: investigation, writing, review and editing. **Marion Leroux**: investigation, writing – review and editing. **Mathieu Moreau**: investigation, validation, writing – review and editing. **Michael Claron**: investigation, writing – review and editing. **Adam Laoufa**: investigation, writing – review and editing. **Agnieszka Kownacka**: investigation, writing – review and editing. **Camille Petitot**: investigation, writing – review and editing. **John Simonet**: investigation, writing – review and editing. **Alexandre M.M. Dias**: investigation, methodology, validation, writing – review and editing. **Sophie Poty**: writing – review and editing. **Charles Truillet**: funding acquisition, supervison, writing – review and editing. **Bertrand Collin**: methodology, resources, validation, writing – review and editing. **Alexandra Oudot**: methodology, resources, validation, writing – review and editing. **Franck Denat**: conceptualization, methodology, supervision, validation, writing – review and editing.

## Funding

This study received funding from the French National Institute of Cancer (INCA) via the project “Keystone” (INCA 16409) and from the European Regional Development Fund via the project “COMETE”.

## Disclosure

Franck Denat is cofounder and shareholder of Chematech.

## Ethics Statement

All animal studies were performed in compliance with preclinical ethical guidelines and approved by the local ethical committee (C2EA Grand Campus, project #47968, Dijon, France).

## Conflicts of Interest

The authors declare no conflicts of interest.

## Supporting information




**Supporting File**: advs76940‐sup‐0001‐SuppMat.docx.

## Data Availability

The data that support the findings of this study are available from the corresponding author upon reasonable request.
