# Supplementary material for: Toward Personalized Theranostics: A Modular PET Platform for Antibody‐Based Imaging and Therapy
Source: Adv Sci (Weinh). 2026 Aug 20;13(62):e76940. doi: 10.1002/advs.76940 (PMC13491523; doi:10.1002/advs.76940)
Supplement: Supplementary file 1 [file ADVS-13-e76940-s001.docx]

Toward Personalized Radiotheranostics: a modular PET platform for patient selection and antibody-based imaging and therapy

*Delphine Vivier^1*^, Megane Grah Kple^1^, Julen Ariztia^1^, Marion Leroux^1^, Mathieu Moreau^1^, Michael Claron^1^, Maxime Cheveau^1^, Adam Laouafa^1,2^, Agnieszka Kownacka^2^, Camille Petitot^2^, John Simonet^2^, Alexandre M. M. Dias^2^, Sophie Poty^1^, Charles Truillet^3^, Bertrand Collin^1,2^, Alexandra Oudot^2^, Franck Denat^1*^*

^1^ Université Bourgogne Europe, CNRS, ICMUB UMR 6302, Dijon, 21000, France

^2^ Université Bourgogne Europe, Centre Georges-François Leclerc-Unicancer, INSERM, BioSanD US 58, Plateforme IMATHERA, 21000 Dijon, France

^3^ Université Paris-Saclay, CEA, CNRS, Inserm, BioMaps, Orsay, 91401, France.

[1.	Abbreviations	2](#_Toc234592322)

[2.	Materials and general procedures	2](#_Toc234592323)

[a.	Reagents	2](#_Toc234592324)

[b.	Instruments (Chemistry)	2](#_Toc234592325)

[c.	Purification and characterization of the bioconjugates	3](#_Toc234592326)

[3.	Probe Synthesis	4](#_Toc234592327)

[a.	Boc-L-Lys(Fmoc)-DFO 2	4](#_Toc234592328)

[b.	DFO-Lys-DBCO 4	4](#_Toc234592329)

[c.	DFO-Lys-DBCO-NCS 4’	5](#_Toc234592330)

[4.	Assessment of metal selectivity of DFO and DOTA via mass spectrometry (cold metalation experiments)	5](#_Toc234592331)

[a.	ZrCl_4_	5](#_Toc234592332)

[b.	LuCl_3_	5](#_Toc234592333)

[5.	Bioconjugation	6](#_Toc234592334)

[a.	d-Tzb 5	6](#_Toc234592335)

[b.	ss-Tzb-DFO-DBCO 6	6](#_Toc234592336)

[c.	ss-Tzb-DFO-DOTAGA 11	7](#_Toc234592337)

[d.	ss-Tzb-DFO-PYTA 12	7](#_Toc234592338)

[e.	ss-Tzb-DFO-MMAE 13	7](#_Toc234592339)

[f.	ss-Tzb-DFO-IR800 14	8](#_Toc234592340)

[g.	ss-Tzb-DFO 15	8](#_Toc234592341)

[h.	ss-Tzb-DOTAGA 16	9](#_Toc234592342)

[i.	N₃-Lu(III)DOTAGA 18	9](#_Toc234592343)

[j.	ss-Tzb-DFO-Lu(III)DOTAGA 17	9](#_Toc234592344)

[k.	ss-Tzb-Zr(IV)DFO-DOTAGA 18	10](#_Toc234592345)

[l.	rd-Tzb-Zr-DFO-DOTAGA 19	10](#_Toc234592346)

[m.	d-rd-Tzb-DFO-DOTAGA 20	10](#_Toc234592347)

[6.	Supplemental figures	11](#_Toc234592348)

[7.	Supplemental tables	27](#_Toc234592349)

# 1. Abbreviations

BLI: Biolayer interferometry; DBCO: Dibenzocyclooctyne; DCM: Dichloromethane; DFO: Deferoxamine; DIPEA: N,N-diisopropylethylamine; DMF: N,N-dimethylformamide; DMSO: Dimethyl sulfoxide; DOL: Degree of labeling; DOTAGA: 2-(4,7,10-tris(carboxymethyl)-1,4,7,10-tetracyclododecan-1-yl)pentanedioic acid; DTPA: Diethylenetriaminepentaacetic acid; EDTA: ethylenediaminetetraacetic acid; ESI: Electrospray ionization; FPLC: Fast protein liquid chromatography; FA: Formic acid; HER2: Human epidermal growth factor receptor 2; HPLC: High performance liquid chromatography; HRMS: High resolution mass spectrometry; ID: Injected dose; IP: Inflection point; iTLC: Instant thin layer chromatography; MS: Mass spectrometry; mTGase: Microbial transglutaminase; p.i.: Post-injection; PBS: Phosphate-buffered saline; PEG: Polyethylene glycol; PNGase F: Peptide-N-glycosidase F; PYTA: 3,6,10,13-tetraaza-1,8(2,6)-dipyridinacyclotetradecaphane-3,6,10,13-tetraacetic acid; RP: Reversed phase; SEC: Size-exclusion chromatography; TFA: Trifluoroacetic acid; TSTU: N,N,N′,N′-Tetramethyl-O-(N-succinimidyl)uronium tetrafluoroborate

# 2. Materials and general procedures

## a. Reagents

All chemicals were acquired from Sigma-Aldrich, Acros Organics, IrisBiotech, MedChemExpress, Licor and Alfa Aesar and used without further purification. DOTAGA derivatives were provided by Chematech (France). Trastuzumab (anti-HER 2 antibody, Herceptin®) was purchased from Roche, purified with a HiTrap Desalting column (GE Healthcare) and concentrated in PBS (pH 7.4) before use. PNGase F was supplied by Promega (USA) in 20 mM Tris-HCl (pH 7.5), 50 mM NaCl and 5 mM EDTA at a concentration of 10 U/µL. mTGase was provided by Zedira (Germany) in PBS (Phosphate Buffer Saline) 0.01 M, pH 7.4, at a concentration of 320 U/mL. All water used was ultra-pure (>18.2 MΩcm^-1^).

## b. Instruments (Chemistry)

NMR

^1^H and ^13^C NMR were recorded either on Bruker Avance Neo 400 MHz (equipped with a 5 mm TBO probe) or Bruker Avance Neo 500 MHz (equipped with a 5 mm BBOF iProbe) spectrometers. NMR spectroscopy chemical shifts are quoted in parts per million relatives to TMS. Spectra calibration was made by using residual signals of partially deuterated solvents summarized in 2010 by Fulmer *et al*. D_2_O and DMSO-*d_6_* were purchased from Eurisotop (subsidiary of Cambridge Isotope Laboratories, Inc.) and raw NMR data were processed with MestReNova software (version 14.3.1-31739).

LCMS analyses

High performance liquid chromatography (HPLC) analyses were performed on an Vanquish system (Thermo Fisher Scientific) equipped with a DAD detector and coupled to a low-resolution mass spectrometry detector MSQ Plus (Thermo Scientific) with an ESI source. Separation was achieved using an RP Kinetex™ column (Phenomenex) (2.6 μm, 100 Å, 50 Å~ 2.1 mm) with HPLC quality solvents: A: H_2_O + 0.1% FA and B: MeCN + 0.1% FA.

Analyses were performed with the following gradient program: 5% to 100% of B in 5 min, 100% B for 1.5 min, 100% to 5% B in 0.1 min and 5% B for 1.9 min, at a flow rate of 0.5 mL/min.

HRMS

High-resolution mass spectra were recorded using a Orbitrap Exploris 240 mass spectrometer (ThermoScientific) equipped with an electrospray ionization source (H-ESI II).

Semi-preparative RP-HPLC

Purifications by semi-preparative HPLC were performed on an automated 1260 Infinity II Preparative LC System (Agilent) equipped with an UV-visible detector, on the following column: BetaBasic-18 column (Thermo Fisher Scientific) (5 μm, 150 Å) at 20 mL/minutes with HPLC grade eluents. Unless specified otherwise, purifications were performed using acetonitrile and water, both supplemented with 0.1% of trifluoroacetic acid or formic acid. The fractions of interest were analyzed by HPLC-MS, pooled, concentrated under reduced pressure to remove organic solvents and freeze-dried.

Freeze-drying

Lyophilization (or freeze-drying) operations were performed with a Christ Alpha 2-4 LD plus.

Radio-analyses

Instant thin-layer chromatography (iTLC) was performed using silicic acid impregnated sheets (iTLC-SA, Agilent) eluted with 0.1 M EDTA pH 5.0 or silica gel impregnated sheets (iTLC-SG, Agilent), eluted with 0.1 M sodium citrate pH 5.0, and analyzed on an AR-2000 radio-TLC plate reader (Bioscan Inc.).

Size-exclusion chromatography (SEC-HPLC) analyses were performed on a HPLC system LC-2000 analytical series (JASCO) or an Ultimate3000 (Thermo Fisher Scientific) equipped with a Superdex 200 Increase 5/150 GL column and a gamma-counter (Bioscan Inc., counting time: 1 min).

## c. Purification and characterization of the bioconjugates

Reactions were followed by reversed phase HPLC (Vanquish, Thermo Fisher Scientific) coupled to a high-resolution Orbitrap mass spectrometry (Exploris 240, Thermo Fisher Scientific) using an ESI source (positive mode). HPLC: A MabPac RP column (2.1 mm x 100 mm, 4µm, Thermo Fisher Scientific) was used at 80°C (denaturing conditions), with a flow rate of 0.5 mL/min and a gradient 20-55% B in 6 min (eluents A: H_2_O + 0.02% TFA + 0.08% FA and B: ACN + 0.02% TFA + 0.08% FA). Mass: Full scan data were acquired at resolving power 30000 at m/z 200 in the m/z range 2200–5000. Data were analyzed with FreeStyle and BioPharma Finder 4.1 software (both Thermo Fisher Scientific). DOL was determined by comparison of native and conjugated antibody masses.

FPLC purification of the conjugates was performed on an Äkta Pure 25 M chromatography system (GE Healthcare Life Sciences) equipped with a HiTrap® MabSelectTM column (MabSelect resin, Protein A, cross-linked agarose, column I.D. 7 mm, bed dimensions 7 x 25 mm, bed volume 1 mL). After deposition of the product, the conjugate was washed with 10 mL of PBS pH 7.4 (1 mL/min). The conjugate was then eluted with 5 mL of 25 mM acetic acid (1 mL/min) and collected in 500 μL fractions. Purification was monitored at 280 and 700 nm.

Concentrations of protein and protein conjugate solutions were determined by measuring the absorbance of the solutions on a Mettler Toledo UV/Vis excellenceUV5Nano spectrometer, at 25 °C. Concentrations of the bioconjugates in mg/mL were calculated by using the extinction coefficient at 280 nm in PBS of trastuzumab (ɛ = 210 000 L.mol^-1^.cm^-1^). For DBCO conjugates, the concentration was calculated using the following formula:

$$[mAb]=\frac{A280-CF \times A309}{\varepsilon}$$

Where:

A280: absorption value of the sample at 280nm

A309: absorption value of the sample at 309 nm

CF: correction factor of DBCO at 280 nm; 1.089.

# 3. Probe Synthesis

## a. Boc-L-Lys(Fmoc)-DFO 2

Boc-L-Lys(Fmoc)-OH (1 equiv., 356 µmol, 167 mg), N,N-diisopropylethylamine (2 equiv., 712 µmol, 124 µL) and N,N,N′,N′-tetramethyl-O-(N-succinimidyl)uronium tetrafluoroborate (1.6 equiv., 570 µmol, 171.7 mg) in 1.5 mL of dry DMSO were stirred at room temperature for 30 min to obtain an orange solution. Deferoxamine mesylate salt (1.1 equiv., 392 µmol, 257 mg) and N,N-diisopropylethylamine (2 equiv., 712 µmol, 124 µL) were then added to the solution. The reaction mixture was stirred at 40°C for 3 hours and purified on semi-preparative HPLC on a BetaBasic-18 column (A: H_2_O 0.1% TFA, B: MeCN 0.1% TFA; with the following gradient program: 2% of B for 8 min, 2 to 30% in 5 min, 30 % to 70% of B in 27 min, at a flow rate of 20 mL/min) to obtain a white solid after lyophilization (m = 170 mg, y = 47%).

RP-HPLC-MS: tr = 4 min, m/z calculated for C_51_H_78_N_8_O_13_ [M+H]+ 1011.58, found 1011.8.

^1^H NMR (500 MHz, DMSO-*d*_6_) δ 9.60 (s, 3H), 7.89 (d, J = 7.5 Hz, 2H), 7.77 (t, J = 5.6 Hz, 2H), 7.73 (t, J = 5.7 Hz, 1H), 7.68 (d, J = 7.5 Hz, 2H), 7.41 (t, J = 7.5 Hz, 2H), 7.33 (t, J = 7.4 Hz, 2H), 7.26 (t, J = 5.7 Hz, 1H), 6.72 (d, J = 8.2 Hz, 1H), 4.28 (d, J = 7.0 Hz, 2H), 4.20 (t, J = 6.9 Hz, 1H), 3.80 (q, J = 7.5 Hz, 1H), 3.48 – 3.41 (m, 6H), 3.09 – 2.90 (m, 8H), 2.57 (t, J = 7.5 Hz, 4H), 2.26 (t, J = 7.4 Hz, 4H), 1.96 (s, 3H), 1.50 (d, J = 11.5 Hz, 8H), 1.37 (d, J = 7.8 Hz, 17H), 1.26 – 1.16 (m, 8H). ^13^C NMR (126 MHz, DMSO-*d*_6_) δ 171.3, 143.9, 140.7, 127.6, 127.0, 125.1, 120.1, 65.2, 47.1, 46.8, 40.1, 39.9, 39.8, 39.6, 38.4, 29.1, 28.8, 28.2, 27.6, 26.0, 23.5, 22.8, 20.3.

## b. DFO-Lys-DBCO 4

Boc-L-Lys(Fmoc)-DFO **2** (1equiv., 55 µmol, 55.6 mg) was dissolved into 1 mL of CH_2_Cl_2_ and 1 mL of TFA and stirred at room temperature for 10 min. After concentration under vacuum, the resulting solution was diluted in 1 mL of dry DMF before the addition of DBCO-NHS (1.05 equiv., 57.8 µmol, 24.8 mg) and N,N-diisopropylethylamine (12 equiv., 660 µmol, 114.7 µL). The reaction mixture was then stirred at room temperature for 1 hour. Finally, 500 µL of diethylamine solution in DMF (2/8) were added and the mixture was stirred at 37°C for 1 hour. After concentration under vacuum, the mixture was purified by semi-preparative HPLC on a BetaBasic-18 column (A: H_2_O 0.1% FA, B: MeCN 0.1% FA; with the following gradient program: 2% of B for 8 min, 2 to 25% in 5 min, 25 % to 65% of B in 27 min, at a flow rate of 20 mL/min) to afford a beige powder (m = 19 mg, y = 34%).

RP-HPLC-MS: t_r_ = 3.25 min, *m/z* calculated for C_52_H_78_N_9_O_11_ [M+H]^+^ 1004.6 found 1004.7.

^1^H NMR (500 MHz, DMSO-*d*_6_) δ 8.39 (bs, 1H), 7.78 (bs, 5H), 7.62 (d, *J* = 7.4 Hz, 1H), 7.59 – 7.53 (m, 1H), 7.53 – 7.48 (m, 1H), 7.48 – 7.42 (m, 2H), 7.39 (td, *J* = 7.5, 1.4 Hz, 1H), 7.34 (td, *J* = 7.5, 1.4 Hz, 1H), 7.29 (d, *J* = 7.4 Hz, 1H), 5.03 (d, *J* = 14.0 Hz, 1H), 4.12 – 4.07 (m, 1H), 3.61 (d, *J* = 14.0 Hz, 1H), 3.48 – 3.40 (m, 8H), 3.05 – 2.91 (m, 6H), 2.68 – 2.61 (m, 2H), 2.57 (t, *J* = 7.4 Hz, 3H), 2.37 – 2.35 (m, 1H), 2.26 (t, *J* = 7.4 Hz, 4H), 2.20 – 2.12 (m, 1H), 1.96 (s, 3H), 1.91 (t, *J* = 7.0 Hz, 2H), 1.78 – 1.69 (m, 1H), 1.59 – 1.45 (m, 7H), 1.44 – 1.32 (m, 9H), 1.32 – 1.12 (m, 13H).

HRMS: *m/z* calculated for C_52_H_78_N_9_O_11_ [M+H]^+^ 1004.58153, found 1004.58009.

## c. DFO-Lys-DBCO-NCS 4’

DFO-Lys-DBCO (1equiv. 2,99 µmol, 30mg) was dissolved into 335 µL of DMF and 8.33 µL of Et_3_N and stirred at room temperature. Then, phenylene diisothiocyanate (1.01 equiv., 3.22µmol, 5.8mg) dissolved in DMF (262µL) was added dropwise and the mixture was stirred for 1h at room temperature. After concentration under vaccum, the mixture was purified by semi-preparative HPLC on a Jupiter C12 column (A: H*2*O 0.1% FA, B: MeCN 0.1% FA; with the following gradient program: 5% of B for 5 min, 5 to 40 % in 5 min, 40% to 85% of B in 30 min, at a flow rate of 15 mL/min) to afford a white powder (m = 9.75 mg, y = 25%).

RP-HPLC-MS: t_r_ = 4.23 min, *m/z* calculated for C_60_H_81_N_11_O_11_S_2_ [M+H]^+^ 1196.5 found 1196.8.

# 4. Assessment of metal selectivity of DFO and DOTA *via* mass spectrometry (cold metalation experiments)

## a. ZrCl_4_

An equimolar solution of DFO-NH_2_ and DOTAGA-PEG_4_-NH_2_ in PBS pH 7.4 was incubated for 30 min at 37°C with 0.2, 0.5, 1 or 2 equivalents of ZrCl_4_. The resulting mixture was then analyzed using reversed phase HPLC (C18 column) coupled to a high-resolution Orbitrap mass spectrometry (Exploris 240, Thermo Fisher Scientific) using an ESI source (positive mode).

## b. LuCl_3_

An equimolar solution of DFO-NH_2_ and DOTAGA-PEG_4_-NH_2_ in ammonium acetate buffer pH 5.0 was incubated for 30 min at 37°C with 0.1, 0.5 or 1 equivalent of LuCl_3_. The resulting mixture was then analyzed using reversed phase HPLC (C18 column) coupled to a high-resolution Orbitrap mass spectrometry (Exploris 240, Thermo Fisher Scientific) using an ESI source (positive mode).

# 5. Bioconjugation

## a. d-Tzb 5

The deglycosylation of Trastuzumab (1 mg) was performed by adding 10 units of recombinant PNGaseF enzyme (Promega) per 1 mg of mAb (1 µL, 10U/µL). The reaction was incubated at 37 °C overnight and then purified via size exclusion chromatography (Sephadex G-25 M, PD-10 column, GE Healthcare) and concentrated using centrifugal filter units with a 50,000 Da molecular weight cut off (Amicon^TM^ Ultra 2 mL Centrifugal Filtration Units, Millipore Corp., Billerica, MA) and phosphate buffered saline (PBS, pH 7.4) to obtain 890 µg of deglycosylated antibody (Figure S4).

## b. ss-Tzb-DFO-DBCO 6

To a solution of d-Tzb **5** (2.65 mg, 250 µL at 10.6 mg/mL in PBS) was added DFO-Lys-DBCO **4** (15 equiv., 4.56 µL at 60mM in PBS), MTGase (3 U/mg of antibody, 331 U/mL, 24 µL) and 250 µL of phosphate buffered saline (PBS, pH 7.4) to adjust the antibody concentration to 5 g/L. The reaction was incubated at 37 °C for 3h and then purified via size exclusion chromatography (Sephadex G-25 M, PD-10 column, GE Healthcare) and concentrated using centrifugal filter units with a 50,000 Da molecular weight cut off (Amicon^TM^ Ultra 2 mL Centrifugal Filtration Units, Millipore Corp., Billerica, MA) and phosphate buffered saline (PBS, pH 7.4) to obtain 2.5 mg of ss-Tzb-DFO-DBCO. The degree of labeling was estimated by mass spectrometry: DOL = 2 (Figure S5).

## c. ss-Tzb-DFO-DOTAGA 11

To a solution of ss-Tzb-DFO-DBCO **6** (2.5 mg, 324 µL at 8.00 mg/mL in PBS) was added N_3_-DOTAGA **7** (10 equiv. *per* DBCO, 11.74 µL at 30 mmol/L in PBS) and the antibody concentration was adjusted to 5 g/L by adding 183 µL of phosphate buffered saline (PBS, pH 7.4). The reaction mixture was stirred at room temperature overnight and purified by Protein A affinity chromatography (Hitrap Mabselect Xtra column,Cytiva) to give 1.9 mg of ss-Tzb-DFO-DOTAGA. The degree of labeling was estimated by mass spectrometry: DOL = 1.8 (Figure S6).

## ss-Tzb-DFO-PYTA 12

To a solution of ss-Tzb-DFO-DBCO **6** (609 µg, 85 µL at 7.17 mg/mL in PBS) was added N_3_-PYTA **8** (10 equiv. *per* accessible DBCO, 8.38 µL at 10mmol/L in DMSO) and the antibody concentration was adjusted to 5 g/L by adding 28.5 µL of phosphate buffered saline (PBS, pH 7.4). The reaction mixture was stirred at room temperature overnight and purified by Protein A affinity chromatography (Hitrap Mabselect Xtra column,Cytiva) to give 346 µg of ss-Tzb-DFO-PYTA. The degree of labeling was measured by mass spectrometry (DOL = 1.8, Figure S7).

## d. ss-Tzb-DFO-MMAE 13

To a solution of ss-Tzb-DFO-DBCO **6** (586 µg, 100 µL at 5.86 mg/mL in PBS) was added N_3_-PEG_4_-vc-PAB-MMAE **9** (BroadPharm, 5 equiv. *per* accessible DBCO, 4 µL at 10 mmol/L in DMSO) and the antibody concentration was adjusted to 5 g/L by adding 13.2 µL of phosphate buffered saline (PBS, pH 7.4). The reaction mixture was stirred at room temperature overnight and purified by Protein A affinity chromatography (Hitrap Mabselect Xtra column,Cytiva) to give 351 µg of ss-Tzb-DFO-MMAE. The degree of labeling was measured by mass spectrometry DOL = 1.8 (Figure S8).

## e. ss-Tzb-DFO-IR800 14

To a solution of ss-Tzb-DFO-DBCO **6** (586 µg, 100 µL at 5.86 mg/mL in PBS) was added N_3_-IR800 **10** (Licor, 10 equiv. *per* accessible DBCO, 4 µL µL at 20 mmol/L in DMSO) and the antibody concentration was adjusted to 5 g/L by adding 13.2 µL of phosphate buffered saline (PBS, pH 7.4). The reaction mixture was stirred at room temperature overnight and purified by Protein A affinity chromatography (Hitrap Mabselect Xtra column, Cytiva) to give 299 µg of ss-Tzb-DFO-IR800. The degree of labeling was measured by mass spectrometry (DOL = 2, Figure S9).

## f. ss-Tzb-DFO 15

To a solution of d-Tzb **5** (1000 µg, 67 µL at 15 mg/mL in PBS) was added DFO-NH_2_ (80 equiv., 36.8 µL at 15 mmol/L in DMSO), MTGase (3 U/mg of antibody, 10 µL) and 36.8 µL of phosphate buffered saline (PBS, pH 7.4) to adjust the antibody concentration to 5 g/L. The reaction mixture was stirred 3 hours at 37°C and purified by Protein A affinity chromatography (Hitrap Mabselect Xtra column,Cytiva) to give 693 µg of ss-Tzb-DFO. The degree of labeling was measured by mass spectrometry (DOL = 2, Figure S13).

## g. ss-Tzb-DOTAGA 16

To a solution of d-Tzb **5** (5.51 mg, 247 µL at 22.32 mg/mL in PBS) was added DOTAGA-PEG_4_-NH_2_ (60 equiv., 151,53 µL at 15 mmol/L in PBS), MTGase (3 U/mg of antibody, 50 µL) and 654 µL of phosphate buffered saline (PBS, pH 7.4) to adjust the antibody concentration to 5 g/L. The reaction mixture was stirred overnight at 37°C and purified by Protein A affinity chromatography (Hitrap Mabselect Xtra column, Cytiva) to give 2.49 mg of ss-Tzb-DOTAGA. The degree of labeling was measured by mass spectrometry (DOL = 1.7, Figure S14).

## h. N₃-Lu(III)DOTAGA 18

To a solution of N_3_-DOTAGA **7** (0.24 µmol, 8 µL at 30 mmol/L in AcONH_4_ pH 5.2) was added LuCl_3_.6H_2_O (5 equiv., 1.2 µmol, 12 µL at 100 mmol/L in AcONH_4_ pH 5.2). The reaction mixture was stirred at 37°C for 45 min before adding disodium EDTA (7 equiv., 7 µL at 250 mmol/L to complex the excess of lutetium (Figure S15).

## i. ss-Tzb-DFO-Lu(III)DOTAGA 17

To a solution of ss-Tzb-DFO-DOTAGA **6** (953 µg, 36 µL at 26.5 mg/mL in PBS) was added N₃-Lu(III) DOTAGA **18** (10 equiv. *per* accessible DBCO, 15 µL at 8.9 mmol/L in AcONH_4_ pH 5.2) and the antibody concentration was adjusted to 5 g/L by adding 140 µL of phosphate buffered saline (PBS, pH 7.4). The reaction mixture was stirred at room temperature overnight and purified by size exclusion chromatography (Superdex 200 Increase 10/300, Cytiva) to give 540 µg of ss-Tzb-DFO-Lu(III)DOTAGA. The degree of labeling was measured by mass spectrometry (DOL = 1.8, Figure S16).

## j. ss-Tzb-Zr(IV)DFO-DOTAGA 18

To a solution of ss-Tzb-DFO-DOTAGA **11** (340 µg, 15 µL at 22.68 mg/mL in PBS) was added ZrCl_4_ (10 equiv., 2.29 µL at 8.9 mmol/L in ACN/H_2_O 50:50) and the antibody concentration was adjusted to 5 g/L by adding 51 µL of phosphate buffered saline (PBS, pH 7.4). The reaction mixture was stirred at 37°C for 2h and purified via size exclusion chromatography (Sephadex G-25 M, Minitrap column, GE Healthcare) and concentrated using centrifugal filter units with a 50,000 Da molecular weight cut off (Amicon^TM^ Ultra 2 mL Centrifugal Filtration Units, Millipore Corp., Billerica, MA) and phosphate buffered saline (PBS, pH 7.4) to give 174 µg of ss-Tzb-Zr(IV)DFO-DOTAGA (Figure S28).

## k. rd-Tzb-Zr-DFO-DOTAGA 19

Because of solubility issue, the SPAAC reaction was performed before the bioconjugation. To a solution of DFO-Lys-DBCO-NCS **4’** (403 µg, 11.25 µL at 30 mM in DMSO) was added DOTAGA-N_3_ (726 µg, 13 µL at 100 mM in DMSO). After 1 hour of incubation at 37°C, 2 mg of trastuzumab was added to the reaction mixture (165.6 µL at 12.1 mg/mL in PBS) and the pH of the solution was adjusted to 9 by adding 810 µL of carbonate/bicarbonate buffer (pH 9, final antibody concentration: 2 g/L). The reaction mixture was incubated at 37°C for 4 hours and then purified via size exclusion chromatography (Sephadex G-25 M, Miditrap column, GE Healthcare) and concentrated using centrifugal filter units with a 50,000 Da molecular weight cut off (Amicon^TM^ Ultra 2 mL Centrifugal Filtration Units, Millipore Corp., Billerica, MA) and phosphate buffered saline (PBS, pH 7.4) to obtain 1.7 mg of rd-Tzb-DFO-DOTAGA **19**. The degree of labeling was estimated by mass spectrometry: DOL = 2.3 (Fig S33).

## l. d-rd-Tzb-DFO-DOTAGA 20

d-rd-Tzb-DFO-DOTAGA **20** was obtained by deglycosylation of rd-Tzb-DFO-DOTAGA **19** with PNGaseF enzyme (10 units of recombinant PNGaseF enzyme (Promega) per 1 mg of mAb (1 µL, 10U/µL)). The reaction was incubated at 37 °C overnight and then purified via size exclusion chromatography (Sephadex G-25 M, PD-10 column, GE Healthcare) and concentrated using centrifugal filter units with a 50,000 Da molecular weight cut off (Amicon^TM^ Ultra 2 mL Centrifugal Filtration Units, Millipore Corp., Billerica, MA) and phosphate buffered saline (PBS, pH 7.4) (Figure S34).

# 6.  Supplemental figures

**Figure S1: HRMS of the DFO-Lys-DBCO 4**

**Figure S2: Selective complexation of Zr^4+^ by DFO.** **A, B.** Deconvoluted mass spectra of DOTAGA and DFO. **C.** Total ion chromatogram of the different mixtures or extracted mass chromatogram of m/z 780 ([DOTAGA-4H^+^+Zr^4+^+H]^+^) and 647 ([DFO-4H^+^+Zr^4+^+H]^+^). **D.** Deconvoluted mass spectrum of [DFO-4H^+^+Zr^4+^+H]^+^. Data were recorded using reverse phase HPLC (C18 column) coupled to a high-resolution Orbitrap mass spectrometer (Exploris 240, Thermo Fisher Scientific) using an ESI source (positive mode) and analyzed using Freestyle software (Thermo Fisher Scientific).

**Figure S3: Selective complexation of Lu^3+^ by DOTAGA.** **A, B.** Deconvoluted mass spectra of DOTAGA and DFO. **C.** Total ion chromatogram of the different mixtures or extracted mass chromatogram of m/z 733 ([DFO-3H^+^+ Lu^3^**^+^**+H]^+^) and 867 ([DOTAGA-3H^+^+ Lu^3^**^+^**+H]^+^). **D.** Deconvoluted mass spectrum of [DOTAGA-3H^+^+ Lu^3^**^+^**+H]^+^. Data were recorded using reverse phase HPLC (C18 column) coupled to a high-resolution Orbitrap mass spectrometer (Exploris 240, Thermo Fisher Scientific) using an ESI source (positive mode) and analyzed using Freestyle software (Thermo Fisher Scientific).

**Figure S4:** **Characterization of d-Tzb 5.** Deconvoluted (**A**) and non deconvoluted (**B**) mass spectra of native Tzb (top) and d-Tzb (bottom). **C.** Total ion (top) and UV at 280 nm (bottom) chromatogram of d-Tzb **5**.

**Figure S5: Characterization of ss-Tzb-DFO-DBCO 6.** Deconvoluted (**A**) and non deconvoluted (**B**) mass spectra of d-Tzb (top) and ss-Tzb-DFO-DBCO (bottom). **C.** Total ion (top) and UV at 280 nm (bottom) chromatogram of ss-Tzb-DFO-DBCO **6**.

**Figure S6:** **Characterization of ss-Tzb-DFO-DOTAGA 11.** Deconvoluted (**A**) and non deconvoluted (**B**) mass spectra of ss-Tzb-DFO-DBCO (top) and ss-Tzb-DFO-DOTAGA (bottom). **C.** Total ion (top) and UV at 280 nm (bottom) chromatogram of ss-Tzb-DFO-DOTAGA **11**

**Figure S7: Characterization of ss-Tzb-DFO-PYTA 12.** Deconvoluted (**A**) and non deconvoluted (**B**) mass spectra of ss-Tzb-DFO-DBCO (top) and ss-Tzb-DFO-PYTA (bottom). **C.** Total ion (top) and UV at 280 nm (bottom) chromatogram of ss-Tzb-DFO-PYTA **12.**

**Figure S8:** **Characterization of ss-Tzb-DFO-MMAE 13.** Deconvoluted (**A**) and non deconvoluted (**B**) mass spectra of ss-Tzb-DFO-DBCO (top) and ss-Tzb-DFO-MMAE (bottom). The peak at 149177 Da corresponds to the degradation of the MMAE, occuring during analysis. **C.** Total ion (top) and UV at 280 nm (bottom) chromatogram of ss-Tzb-DFO-MMAE **13**.

**Figure S9: Characterization of ss-Tzb-DFO-IR800 14.** Deconvoluted (**A**) and non deconvoluted (**B**) mass spectra of ss-Tzb-DFO-DBCO (top) and ss-Tzb-DFO-IR800 (bottom). **C.** Total ion (top) and UV at 280 nm (bottom) chromatogram of ss-Tzb-DFO-IR800 **14**.

**Figure S10:** **Thermal stability of the different bimodal conjugates**. The melting temperatures (Tm) of the various antibody domains are reported, along with the onset temperature of CH2 domain unfolding and the temperature of aggregation onset (T_turbidity_).

**Figure S11: Size-exclusion chromatography of the different conjugates.**

**Figure S12:** **Synthesis of the different control immunoconjugates**

**Figure S13: Characterization of ss-Tzb-DFO 15.** Deconvoluted (**A**) and non deconvoluted (**B**) mass spectra of d-Tzb (top) and ss-Tzb-DFO (bottom). **C.** Total ion (top) and UV at 280 nm (bottom) chromatogram of ss-Tzb-DFO **15**.

**Figure S14: Characterization of ss-Tzb-DOTAGA 16.** Deconvoluted (**A**) and non deconvoluted (**B**) mass spectra of d-Tzb (top) and ss-Tzb-DOTAGA (bottom). **C.** Total ion (top) and UV at 280 nm (bottom) chromatogram of ss-Tzb-DOTAGA **16**.

**Figure S15:** **Characterization of N_3_-Lu(III)DOTAGA 18.** UV chromatogram (214 nm, left) and mass spectrum (right).

**Figure S16: Characterization of ss-Tzb-DFO-Lu(III)DOTAGA 17.** Deconvoluted (**A**) and non deconvoluted (**B**) mass spectra of ss-Tzb-DFO-DBCO (top) and ss-Tzb-DFO-Lu(III)DOTAGA (bottom). **C.** Total ion (top) and UV at 280 nm (bottom) chromatogram of ss-Tzb-DFO-Lu(III)DOTAGA **17**.

**Figure S17:** **Thermal stability of bischelate conjugates and control constructs**. The melting temperatures (Tm) of the various antibody domains are reported, along with the onset temperature of CH2 domain unfolding and the temperature of aggregation onset (T_turbidity_).

**Figure S18: Kinetic curves** obtained with HER2 receptor for d-Tzb (**A**), ss-Tzb-DFO-DBCO **6** (**B**), ss-Tzb-DFO-DOTAGA **11** (**C**), ss-Tzb-DFO-Lu(III)DOTAGA **17** (**D**), ss-Tzb-(Zr(IV)DFO-DOTAGA **18** (**E**), **and dissociation constants** (**F**).

**Figure S19: Stability of [^89^Zr]Zr-conjugates in PBS with 1,000 equivalents of ethylenediaminetetraacetic acid (EDTA) or 1,000 equivalents of DFO, measured by iTLC or HPLC.** Error bars represent standard deviations (n=3). **p < 0.01, ***p < 0.005.

**Figure S20:** **Stability of [^89^Zr]Zr-conjugates in human plasma - Formation of High-Molecular Weight species.**

**Figure S21: Stability of [^177^Lu]Lu-conjugates in PBS with 1,000 equivalents of ethylenediaminetetraacetic acid (EDTA), measured by iTLC.** Error bars represent standard deviations (n=3). **p < 0.01, ***p < 0.005.

**Figure S22: ^89^Zr radiolabeling quality control. A**. iTLC of [^89^Zr]Zr-ss-Tzb-DFO **15** (top), [^89^Zr]Zr-ss-Tzb-DFO-DOTAGA **11** (middle) and [^89^Zr]Zr-ss-Tzb-DFO-Lu(III)DOTAGA **17** (bottom) before purification. **B.** UV chromatogram (top) and radiotrace (bottom) of [^89^Zr]Zr-ss-Tzb-DFO **15** (left), [^89^Zr]Zr-ss-Tzb-DFO-DOTAGA **11** (middle) and [^89^Zr]Zr-ss-Tzb-DFO-Lu(III)DOTAGA **17** (right) before purification.

**Figure S23: ^177^Lu radiolabeling quality control. A**. iTLC of [^177^Lu]Lu-ss-Tzb-DOTAGA **16** (top) and [^177^Lu]Lu-ss-Tzb-DFO-DOTAGA **11** (bottom) before injection. **B.** UV chromatogram (top) and radiotrace (bottom) of [^177^Lu]Lu-ss-Tzb-DFO-DOTAGA **11**.

**Figure S24: Stability of [^89^Zr]Zr-conjugates in PBS and human plasma at 80 MBq/mg.** Error bars represent standard deviations (n=3).

**Figure S25: Stability of [^177^Lu]Lu-ss-Tzb-DFO-DOTAGA in PBS and plasma, measured by HPLC.** Error bars represent standard deviations (n=3).

**Figure S26: Injected doses of the different conjugates in MBq.**

**Figure S27:** **Biodistribution of [^177^Lu]Lu-Tzb-DFO-DOTAGA 11 and [^177^Lu]Lu-Tzb-DOTAGA 16. A.** Ex vivo biodistribution (n=3) of **11** and **16**. **B.** Pharmacokinetics of tumor uptake.

**Figure S28: Characterization of ss-Tzb-Zr(IV)DFO-DOTAGA 18.** Deconvoluted (**A**) and non deconvoluted (**B**) mass spectra of ss-Tzb-DFO-DOTAGA (top) and ss-Tzb-Zr(IV)DFO-DOTAGA (bottom).

**Figure S29 : Kinetic curves** obtained with mFcRn for native Tzb (**A**), d-Tzb (**B**), ss-Tzb-DFO-DBCO **6** (**C**), ss-Tzb-DFO-DOTAGA **11** (**D**), ss-Tzb-DFO-Lu(III)DOTAGA **17** (**E**), ss-Tzb-Zr(IV)DFO-DOTAGA **18** (**F**), **and dissociation constants** (**G**).

**Figure S30 : Stability of [^177^Lu]Lu-ss-Tzb-DFO-DOTAGA and [^177^Lu]Lu-ss-Tzb-DOTAGA-DFO (reverse bischelate) in PBS and plasma, measured by HPLC.** Error bars represent standard deviations (n=3).

**Figure S31: Hydrophobic interaction chromatograms of ss-Tzb-DFO-DOTAGA 11, ss-Tzb-Zr(IV)DFO-DOTAGA and ss-Tzb-DFO-Lu(III)DOTAGA, together with co-injection of the latter constructs**, monitored at 280nm (column : MabPac HIC-butyl concentration : 1mg/mL, flow rate: 1mL.min^-1^).

**Figure S32 : Internalization study of ss-Tzb-DFO-DOTAGA 11, rd-Tzb-DFO-DOTAGA 19 and d-rd-Tzb-DFO-DOTAGA 20 following ^89^Zr or ^177^Lu radiolabeling.** The intracellular fraction (**A**) and membrane-bound fraction (**B**) were determined after 4 and 24h of incubation with HCC1954 cells. Blocking experiments were conducted using a 100-fold excess of non-radiolabeled trastuzumab.

**Figure S33: Characterization of rd-Tzb- DFO-DOTAGA 19.** Deconvoluted (**A**) and non deconvoluted (**B**) mass spectra of native trastuzumab (top) and rd-Tzb-DFO-DOTAGA (bottom). **C.** Total ion (top) and UV at 280 nm (bottom) chromatogram of rd-Tzb-DFO-DOTAGA **19**.

**Figure S34:** **Characterization of d-rd-Tzb- DFO-DOTAGA 20.** Deconvoluted (**A**) and non deconvoluted (**B**) mass spectra of rd-Tzb-DFO-DOTAGA (top) and d-rd-Tzb-DFO-DOTAGA (bottom). **C.** Total ion (top) and UV at 280 nm (bottom) chromatogram of d-rd-Tzb-DFO-DOTAGA **20**.

# 7. Supplemental tables

**Table S1: Ex vivo distribution of [⁸⁹Zr]Zr-Tzb-DFO-DOTAGA 11, [⁸⁹Zr]Zr-Tzb-DFO 15 and [⁸⁹Zr]Zr-Tzb-DFO-Lu(III)DOTAGA 17.** Values are expressed as the percentage of injected dose per gram of tissue (%ID/g ± SD).

**Table S2:** **Ex vivo distribution of [^177^Lu]Lu-Tzb-DFO-DOTAGA 11 and [^177^Lu]Lu-Tzb-DOTAGA 16.** Values are expressed as the percentage of injected dose per gram of tissue (%ID/g ± SD).

**Table S3: Tumor to organ ratios of [⁸⁹Zr]Zr-Tzb-DFO-DOTAGA 11, [⁸⁹Zr]Zr-Tzb-DFO 15 and [⁸⁹Zr]Zr-Tzb-DFO-Lu(III)DOTAGA 17**

**Table S4: Tumor to organ ratios of [^177^Lu]Lu-Tzb-DFO-DOTAGA 11 and [^177^Lu]Lu-Tzb-DOTAGA 16.**

**Table S5: Therapy study. A.** Injected doses in MBq and **B.** Tumor volumes at injection.

**Table S6: Body weight of mice in control and treated groups during radioimmunotherapy.** Data are expressed in grams.

**Table S7:** **Tumor volumes of mice in control and treated groups during radioimmunotherapy.** Data are expressed in mm^3^.
